# Cardiovascular and mortality outcomes with GLP-1 receptor agonists vs other glucose-lowering drugs in individuals with NAFLD and type 2 diabetes: a large population-based matched cohort study

**DOI:** 10.1007/s00125-023-06057-5

**Published:** 2023-12-20

**Authors:** Arunkumar Krishnan, Carolin V. Schneider, Yousaf Hadi, Diptasree Mukherjee, Bandar AlShehri, Saleh A. Alqahtani

**Affiliations:** 1 Department of Supportive Oncology, Levine Cancer Institute, Atrium Health, Charlotte, NC USA; 2 Division of Gastroenterology and Hepatology, Johns Hopkins University School of Medicine, Baltimore, MD USA; 3 Department of Medicine, Section of Gastroenterology and Hepatology, West Virginia University School of Medicine, Morgantown, WV USA; 4 Department of Medicine III, Gastroenterology, Metabolic Diseases, and Intensive Care, University Hospital RWTH Aachen, Aachen, Germany; 5 Department of Medicine, Apex Institute of Medical Science, Kolkata, West Bengal India; 6 Diabetes and Endocrinology, Faculty of Medicine, University of Jeddah, Jeddah, Saudi Arabia; 7 Liver Transplant Center, King Faisal Specialist Hospital and Research Center, Riyadh, Saudi Arabia

**Keywords:** Adverse cardiovascular events, GLP-1RA, Glucagon-like peptide-1 receptor agonists, Mortality, NAFLD, Non-alcoholic fatty liver disease, Outcomes, SGLT2i, Sodium–glucose cotransporter 2 inhibitors, T2DM, Type 2 diabetes mellitus

## Abstract

**Aims/hypothesis:**

We aimed to determine whether the use of glucagon-like peptide-1 receptor agonists (GLP-1RA) in individuals with non-alcoholic fatty liver disease (NAFLD) and type 2 diabetes mellitus decreases the risk of new-onset adverse cardiovascular events (CVEs) and mortality rate compared with other glucose-lowering drugs in a real setting at a population level.

**Methods:**

We conducted a population-based propensity-matched retrospective cohort study using TriNetX. The cohort comprised patients over 20 years old who were newly treated with glucose-lowering drugs between 1 January 2013 and 31 December 2021, and followed until 30 September 2022. New users of GLP-1RAs were matched based on age, demographics, comorbidities and medication use by using 1:1 propensity matching with other glucose-lowering drugs. The primary outcome was the new onset of adverse CVEs, including heart failure, composite incidence of major adverse cardiovascular events (MACE; defined as unstable angina, myocardial infarction, or coronary artery procedures or surgeries) and composite cerebrovascular events (defined as the first occurrence of stroke, transient ischaemic attack, cerebral infarction, carotid intervention or surgery), and the secondary outcome was all-cause mortality. Cox proportional hazards models were used to estimate HRs.

**Results:**

The study involved 2,835,398 patients with both NAFLD and type 2 diabetes. When compared with the sodium–glucose cotransporter 2 (SGLT2) inhibitors group, the GLP-1RAs group showed no evidence of a difference in terms of new-onset heart failure (HR 0.97; 95% CI 0.93, 1.01), MACE (HR 0.95; 95% CI 0.90, 1.01) and cerebrovascular events (HR 0.99; 95% CI 0.94, 1.03). Furthermore, the two groups had no evidence of a difference in mortality rate (HR 1.06; 95% CI 0.97, 1.15). Similar results were observed across sensitivity analyses. Compared with other second- or third-line glucose-lowering medications, the GLP-1RAs demonstrated a lower rate of adverse CVEs, including heart failure (HR 0.88; 95% CI 0.85, 0.92), MACE (HR 0.89; 95% CI 0.85, 0.94), cerebrovascular events (HR 0.93; 95% CI 0.89, 0.96) and all-cause mortality rate (HR 0.70; 95% CI 0.66, 0.75).

**Conclusions/interpretation:**

In individuals with NAFLD and type 2 diabetes, GLP-1RAs are associated with lower incidences of adverse CVEs and all-cause mortality compared with metformin or other second- and third-line glucose-lowering medications. However, there was no significant difference in adverse CVEs or all-cause mortality when compared with those taking SGLT2 inhibitors.

**Graphical Abstract:**

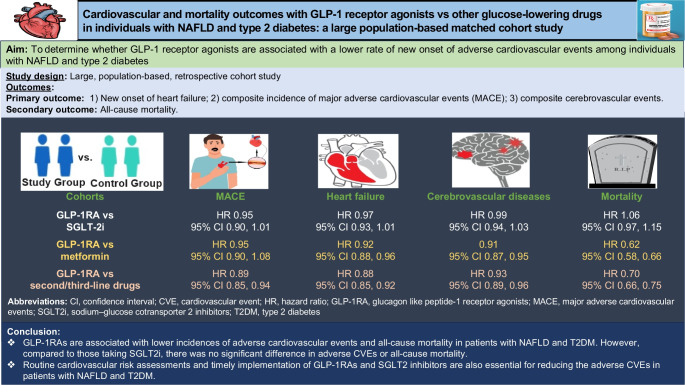

**Supplementary Information:**

The online version contains peer-reviewed but unedited supplementary material available at 10.1007/s00125-023-06057-5.


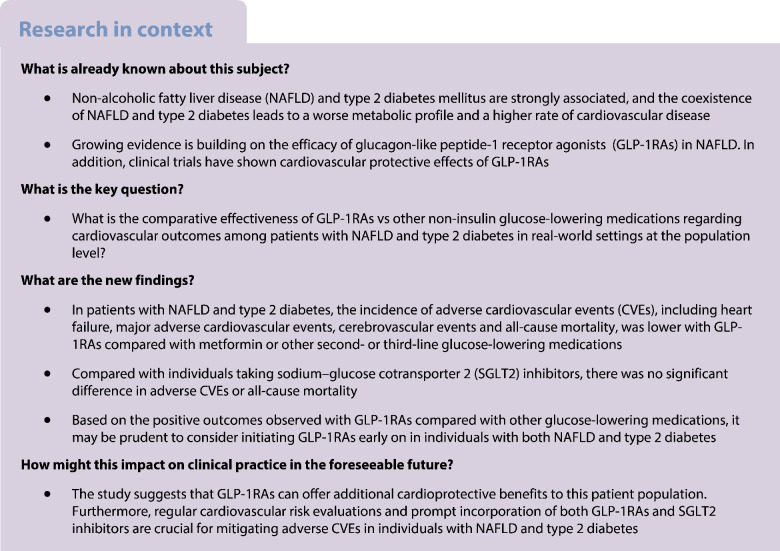


## Introduction

Non-alcoholic fatty liver disease (NAFLD) is considered to be the hepatic manifestation of the metabolic syndrome, which is believed to affect approximately 25% of adults worldwide and more than half of individuals with type 2 diabetes mellitus [1]. Studies have shown that there is a bidirectional relationship between NAFLD and type 2 diabetes, and that NAFLD may precede and/or promote the development of type 2 diabetes [2, 3]. NAFLD and type 2 diabetes coexist in clinical practice and act synergistically to drive adverse outcomes [4]. The presence of NAFLD increases the incidence of type 2 diabetes and accelerates the development of complications in type 2 diabetes [5]. Moreover, the coexistence of NAFLD and type 2 diabetes leads to a worse metabolic profile and higher cardiovascular risk [6, 7]. NAFLD is currently treated by reducing weight and improving insulin resistance through lifestyle interventions [8]. However, lifestyle changes are difficult to maintain over the long term [9].

The use of glucagon-like peptide-1 receptor agonists (GLP-1RAs), a class of drugs approved for treating type 2 diabetes, has been explored in patients with NAFLD [10]. GLP-1RAs are one of the few glucose-lowering medications that result in weight loss, which has a robust association with improvement of hepatic steatosis [11]. In addition to this, GLP-1RAs may directly improve hepatic steatosis via the upregulation of fatty acid metabolism and insulin signalling pathways [12]. Notably, growing evidence is building about the efficacy of GLP-1RAs in NAFLD [13]. It has also been reported that GLP-1RAs may have cardiovascular protective effects among individuals with type 2 diabetes, and they therefore provide an attractive therapy option for patients with NAFLD [14]. However, no head-to-head comparisons of GLP-1RAs and other non-insulin glucose-lowering medications exist for patients with NAFLD and type 2 diabetes. It is unclear whether using one drug over another yields differences in cardiovascular outcomes. Therefore, we aimed to determine whether GLP-1RAs, compared with sodium–glucose cotransporter 2 (SGLT2) inhibitors, metformin or other second- or third-line glucose-lowering medications, are associated with a lower rate of new-onset adverse cardiovascular events (CVEs) among patients with NAFLD and type 2 diabetes.

## Methods

### Study design and data source

This large, population-based, retrospective cohort study was conducted using the TriNetX research network (Cambridge, MA, USA). TriNetX is a federated multicentre research network that provides real-time access to an anonymised dataset from participating healthcare organisations' electronic health records (EHR). Details of the data source, quality checks and diagnosis codes used for patient selection (according to predefined ICD-9 [http://www.icd9data.com/2007/Volume1/default.htm] and ICD-10 [https://icd.who.int/browse10/2019/en] codes) are described in the electronic supplementary material (ESM) Methods. Details of the TriNetX network are described in previous studies [15, 16]. We followed the Strengthening the Reporting of Observational Studies in Epidemiology (STROBE) reporting guideline.

### Study participants

We identified all adult (aged ≥20 years) patients with NAFLD and type 2 diabetes who had newly started treatment with non-insulin glucose-lowering drugs (metformin, GLP-1RAs, SGLT2 inhibitors, sulfonylureas, meglitinides, thiazolidinediones, acarbose or dipeptidyl peptidase-4 inhibitors) between 1 January 2013 and 31 December 2021. We limited the study cohort to patients with at least 1 year of follow-up before cohort entry (i.e. patients who received their first glucose-lowering prescription more than 1 year before cohort entry). Furthermore, to reduce reverse causality and detection bias, we only included individuals with more than 1 year of follow-up after the start of the study.

The identification of NAFLD at the study's outset was based on the presence of specific ICD‐9 and ICD-10 codes. Individuals diagnosed with other liver diseases with any ICD codes were excluded from the NAFLD group. Individuals were excluded if they met any of the following criteria: chronic liver disease other than NAFLD, including alcohol, viral, drug-induced, autoimmune and genetic diseases, liver cirrhosis, or clinical diagnosis of hepatic decompensation (such as oesophageal varices or ascites); history of excessive alcohol use, alcohol abuse, or alcohol use disorder or history of alcohol-related disorders; HIV infection; solid organ transplantation; or individuals with eGFR <30 ml/min per 1.73m^2^ (within 6 months before the cohort entry) and history of undergoing dialysis treatment. Finally, individuals with a history of heart failure, ischaemic heart disease, unstable angina, myocardial infarction, aortic aneurysm or dissection, stroke (ischaemic or haemorrhagic stroke), cerebral infarction, transient ischaemic attack, carotid intervention or surgery, coronary stenting, percutaneous coronary intervention (PCI) or coronary artery bypass before inclusion to the cohort or prior to the index event were also excluded. We further excluded individuals with a history of treatment with insulin before their initial prescription for a non-insulin glucose-lowering drug (since insulin therapy at baseline is considered for patients with advanced disease), women with a history of polycystic ovary syndrome or gestational diabetes, and individuals with type 1 diabetes. Patients were followed from 1 year after cohort entry until a diagnosis of incident adverse CVEs, switch to a comparator drug, any cause of death or the end of the study (30 September 2022), whichever occurred first.

### Drug exposure

The study cohort consisted of new users of GLP-1RAs (dulaglutide, exenatide, liraglutide, lixisenatide or semaglutide), SGLT2 inhibitors (canagliflozin, dapagliflozin or empagliflozin), metformin and other second- or third-line glucose-lowering drugs (thiazolidinediones, dipeptidyl peptidase-4 inhibitors, sulfonylureas, meglitinides, α-glucosidase inhibitors, insulin or a combination of glucose-lowering drugs). Furthermore, the study cohort included individuals who either switched to or added on treatment with other glucose-lowering drugs. Cohort entry was defined as the date of the first-ever prescription for one of the drugs of interest (GLP-1RAs, SGLT2 inhibitors, metformin or other second- or third-line glucose-lowering drugs) during the study period, and their respective first exposure was defined as the index event. We used a lag of 6 months for all exposures to minimise protopathic bias and allow for a minimum and sufficient latency period after cohort entry [17, 18].

### Matching process

We used a propensity score matching (PSM) method to compare the new users of GLP-1RAs with new users of SGLT2 inhibitors, metformin and other second- or third-line glucose-lowering drugs. The PSM was performed using 1:1 to reduce the confounding effects. The covariates were adjusted in the PSM model for a priori-identified potential confounders, such as age, sex, race/ethnicity, nicotine dependence, BMI, type 2 diabetes, hypertension, hyperlipidaemia, hypercholesterolaemia, chronic respiratory disease, chronic renal diseases, BP, chronic kidney disease, peripheral vascular diseases, diabetes-related microvascular complications, glomerular diseases, cirrhosis of the liver, osteoporosis, sleep apnoea, abnormal laboratory findings (HbA_1c_, serum cholesterol, LDL-cholesterol, HDL-cholesterol and triacylglycerol [TG]) and intake of cardiovascular medications (Table 1). Logistic regression was performed to obtain the propensity scores, and a greedy nearest-neighbour matching algorithm was used to perform the matching with a calliper of 0.1 pooled SD. The balancing of potential confounding variables was evaluated using standardised mean differences (SMD) with a threshold set a priori at 0.10. We used SMD to measure the magnitude of difference between the groups rather than the *p* value because of their insensitivity to sample size. Logistic regression was performed using both Python (Python Software Foundation, Wilmington, Delaware, USA) and R 3.4.4 software (R Foundation for Statistical Computing, Vienna, Austria) to ensure the outputs matched, and the order of the rows in the covariate matrix was randomised to eliminate this bias.

**Table 1:** Baseline characteristics of individuals with NAFLD and type 2 diabetes who are new users of GLP-1RAs vs matched new users of SGLT2 inhibitors

Variables	Before the PSM			After the PSM		
	GLP-1RAs (*N* =53,249)	SGLT2 inhibitors (*N* =39,795)	SMD	GLP-1RAs (*N* =38,804)	SGLT2 inhibitors (*N* =38,804)	SMD
Age (years), mean±SD	54.6±12	56.3±11.5	0.1460	56.1±11.5	56.1±11.5	0.0030
Sex (female), *n* (%)	32,188 (60.4)	20,204 (50.8)	0.1957	20,144 (51.9)	20,123 (51.9)	0.0011
Ethnicity, *n* (%)						
Hispanic or Latino	5161 (9.7)	4131 (10.4)	0.0229	3943 (10.2)	3992 (10.3)	0.0042
Race, *n* (%)						
White	38,990 (73.2)	29,151 (73.3)	0.0007	28,693 (73.9)	28,532 (73.5)	0.0094
Black or African Americans	7649 (14.4)	4839 (12.2)	0.0650	4655 (12.0)	4766 (12.3)	0.0088
Other	5301 (10.0)	4563 (11.5)	0.0489	4315 (11.1)	4353 (11.2)	0.0031
Nicotine dependence, *n* (%)	9492 (17.8)	7051 (17.7)	0.0028	6697 (17.3)	6839 (17.6)	0.0096
BMI (kg/m^2^), mean±SD	37.1±6.85	35.5±6.78	0.2368	36.7±6.78	35.6±6.78	0.1713
Comorbidities, *n* (%)						
Hypertension	40,674 (76.4)	30,591 (76.9)	0.0115	29,852 (76.9)	29,819 (76.8)	0.0020
Hyperlipidaemia	32,759 (61.5)	24,980 (62.8)	0.0258	24,471 (63.1)	24,296 (62.6)	0.0093
Chronic lower respiratory diseases	17,894 (33.6)	12,345 (31.0)	0.0553	11,960 (30.8)	12,045 (31.0)	0.0047
Hypercholesterolaemia	12,968 (24.4)	10,016 (25.2)	0.0189	9727 (25.1)	9735 (25.1)	0.0005
Diabetic polyneuropathy	9289 (17.4)	6783 (17.0)	0.0106	6745 (17.4)	6646 (17.1)	0.0068
Chronic kidney disease	7534 (14.1)	5698 (14.3)	0.0049	5414 (14.0)	5474 (14.1)	0.0045
Peripheral vascular diseases	4094 (7.7)	3170 (8.0)	0.0103	2988 (7.7)	3035 (7.8)	0.0045
Diabetic retinopathy	3629 (6.8)	2614 (6.6)	0.0099	2582 (6.7)	2560 (6.6)	0.0023
Diabetic nephropathy	3795 (7.1)	2901 (7.3)	0.0063	2839 (7.3)	2826 (7.3)	0.0013
Glomerular diseases	1841 (3.5)	1251 (3.1)	0.0176	1303 (3.4)	1222 (3.1)	0.0118
Cirrhosis of liver	4017 (7.5)	3508 (8.8)	0.0464	3013 (7.8)	3400 (8.8)	0.0362
Osteoporosis	2514 (4.7)	1860 (4.7)	0.0022	1768 (4.6)	1825 (4.7)	0.0070
Obstructive sleep apnoea	18,993 (35.7)	12,771 (32.1)	0.0790	12,423 (32.0)	12,532 (32.3)	0.0057
Cardiovascular medications, *n* (%)						
β-blockers	23,660 (44.4)	18,487 (46.5)	0.0406	17,651 (45.5)	17,755 (45.8)	0.0054
Antiarrhythmics	25,198 (47.3)	18,420 (46.3)	0.0207	18,130 (46.7)	17,831 (46.0)	0.0155
Antilipaemic agents	36,367 (68.3)	28,627 (71.9)	0.0796	27,986 (72.1)	27,733 (71.5)	0.0145
ACE inhibitors	25,681 (48.2)	19,787 (49.7)	0.0299	19,378 (49.9)	19,212 (49.5)	0.0086
Angiotensin II inhibitors	15,452 (29.0)	12,303 (30.9)	0.0414	11,735 (30.2)	11,728 (30.2)	0.0004
Diuretics	26,229 (49.3)	19,194 (48.2)	0.0205	19,191 (49.5)	18,549 (47.8)	0.0331
Vitamin D supplement	16,968 (31.9)	11,772 (29.6)	0.0495	11,923 (30.7)	11,476 (29.6)	0.0251
Vitamin E supplement	2647 (5.0)	2015 (5.1)	0.0042	1950 (5.0)	1950 (5.0)	<0.0001
Calcium channel blockers	15,856 (29.8)	12,458 (31.3)	0.0332	11,871 (30.6)	12,066 (31.1)	0.0109
Antihypertensives, other	11,075 (20.8)	8761 (22.0)	0.0297	8369 (21.6)	8435 (21.7)	0.0041
Antihypertensive combinations	325 (0.6)	682 (1.7)	0.1031	323 (0.8)	340 (0.9)	0.0048
Glucose-lowering medications, *n* (%)						
Metformin	39,159 (73.5)	29,625 (74.4)	0.0206	28,675 (73.9)	28,933 (74.6)	0.0152
Insulin	28,667 (53.8)	20,754 (52.2)	0.0337	21,048 (54.2)	20,178 (52.0)	0.0449
Glipizide	11,358 (21.3)	8733 (21.9)	0.0149	8434 (21.7)	8521 (22.0)	0.0054
Sitagliptin	10,964 (20.6)	9599 (24.1)	0.0848	8386 (21.6)	9380 (24.2)	0.0610
Empagliflozin	5763 (10.8)	6515 (16.4)	0.1624	5014 (12.9)	6316 (16.3)	0.0951
Pioglitazone	4878 (9.2)	3869 (9.7)	0.0192	3745 (9.7)	3796 (9.8)	0.0044
Canagliflozin	3935 (7.4)	4757 (11.9)	0.1549	3418 (8.8)	4672 (12.0)	0.1059
Glyburide^a^	3364 (6.3)	2480 (6.2)	0.0035	2525 (6.5)	2435 (6.3)	0.0095
Dapagliflozin	2498 (4.7)	3011 (7.6)	0.1201	2228 (5.7)	2913 (7.5)	0.0710
Linagliptin	2539 (4.8)	2436 (6.1)	0.0597	1972 (5.1)	2371 (6.1)	0.0447
Repaglinide	702 (1.3)	545 (1.4)	0.0044	513 (1.3)	530 (1.4)	0.0038
Rosiglitazone	499 (0.9)	282 (0.7)	0.0253	270 (0.7)	279 (0.7)	0.0028
Acarbose	334 (0.6)	280 (0.7)	0.0094	264 (0.7)	265 (0.7)	0.0003
Nateglinide	271 (0.5)	228 (0.6)	0.0087	224 (0.6)	213 (0.5)	0.0038
Ertugliflozin	210 (0.4)	208 (0.5)	0.0190	198 (0.5)	192 (0.5)	0.0022
Alogliptin	452 (0.8)	362 (0.9)	0.0065	363 (0.9)	352 (0.9)	0.0030

Data are presented as mean±SD or *n* (%)

^a^Glibenclamide is known as glyburide in the USA and Canada

### Outcome

The primary outcome was to assess the incidence of new-onset adverse CVEs, which were categorised as: (1) heart failure; (2) major adverse cardiovascular events (MACE); and (3) composite cerebrovascular events. MACE, as a composite endpoint, was defined as the first occurrence of unstable angina, myocardial infarction or revascularisation (including PCI or coronary artery bypass graft) [19, 20]. A composite of cerebrovascular events was defined as the first occurrence of stroke (haemorrhagic or ischaemic stroke), transient ischaemic attack, cerebral infarction, carotid intervention or surgery [19, 20]. The secondary outcome was to evaluate the incidence of all-cause mortality.

### Statistical analyses

All analyses were performed using the TriNetX real-time analytics platform. This approach involves dynamic and immediate data analysis, enabling continuous processing and interpretation of data as it is generated. Categorical variables were compared using the Pearson χ^2^ test, and continuous variables were compared using an independent-sample *t* test. Continuous variables were expressed as mean ± SD and categorical variables were presented as frequency and percentage. Analyses were performed to examine the rate of adverse CVEs using Cox proportional hazards models. HRs and CIs, along with tests for proportionality, were calculated using R's Survival package v3.2-3. The results were validated by comparing them with the output from SAS version 9.4. Patients were censored when the time window ended or the day after the last fact in their record. We utilised a 1:1 propensity matching strategy to establish comparable groups of patients treated with different glucose-lowering drugs, including GLP-1RA, SGLT2 inhibitors and metformin. In addition, we used this matching approach to balance the covariates between the groups effectively. To account for clustering within the 1:1 propensity-matched sample and address the loss of independence among individuals resulting from the matching procedure, we incorporated a robust variance estimator in the Cox regression model [21]. The robust variance estimator is essential in enhancing the accuracy of our analytical approach and ensuring the validity of the study's findings. A priori-defined two-sided alpha of <0.05 was used for statistical significance.

### Ancillary analysis

We used metformin as a control in the secondary analysis. In this analysis, we matched new users of GLP-1RAs to new metformin users on propensity scores.

### Sensitivity analyses

We conducted two sensitivity analyses to ensure the robustness of our findings due to the heterogeneous nature of adverse CVEs. The first sensitivity analysis evaluated the influence of different reference groups/disease stages. We compared the use of GLP-1RAs with second- or third-line glucose-lowering therapy to minimise confounding [22], thereby allowing us to explore the impact of including a reference group with the same disease stage, even though we adjusted for microvascular complications. In the second sensitivity analysis, we estimated the rates of new incidences of study outcomes by excluding patients with outcomes 2 years after the index event. Both analyses were performed using the same methods as the primary analysis.

## Results

### Baseline characteristics (GLP-1RAs vs SGLT2 inhibitors)

**Fig. 1:**
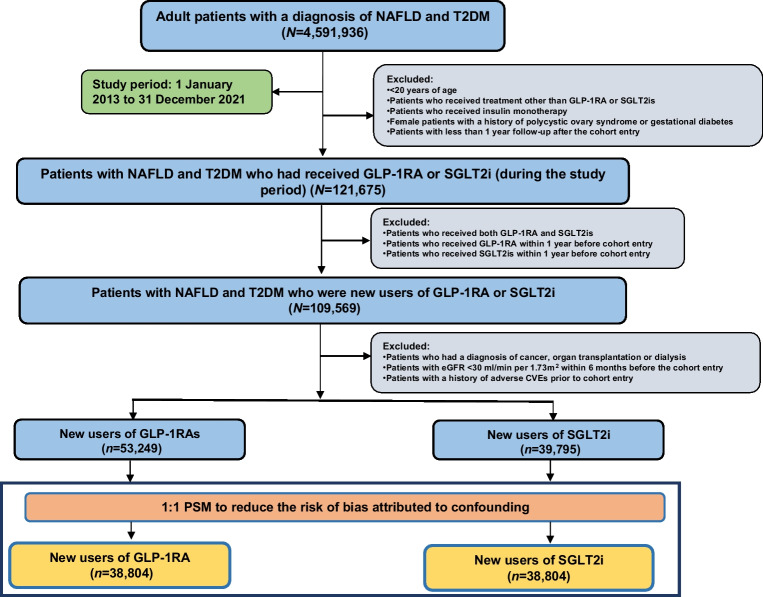
Flow chart of patient selection in the study cohort for new users of GLP-1RAs and new users of SGLT2 inhibitors (active comparator). SGLT2i, SGLT2 inhibitor; T2DM, type 2 diabetes mellitus

A total of 4,591,936 individuals with NAFLD and type 2 diabetes were identified. Of those, 53,249 individuals had newly received GLP-1RAs (GLP-1RAs group) and 39,795 had newly received SGLT2 inhibitors (control group; Fig. 1). Patients receiving GLP-1RAs were on average younger than patients in the control group (54.6 vs 56.3 years). Individuals in both groups were more likely to be female and white. In the GLP-1RAs cohort, BMI was higher (mean [SD], 37.1 [6.85] vs 35.5 [6.78]), and individuals were more likely to have a history of sleep apnoea (Table 1).

The mean follow-up was 4.8±1.1 years for the GLP-1RAs group and 5.3±1.2 years for the SGLT2 inhibitors group. After PSM, the GLP-1RAs and SGLT2 inhibitors groups (*n*=38,804 in each) were well matched (ESM Fig. 1). Clinical characteristics of the GLP-1RAs and SGLT2 inhibitors groups are compared in ESM Table 1. After PSM, among the lipid profiles, TG were significantly higher among the SGLT2 inhibitors group (2.44 ± 2.84 mmol/l vs 2.51 ± 2.81 mmol/l, ESM Table 1).

### Outcomes

**Fig. 2:**
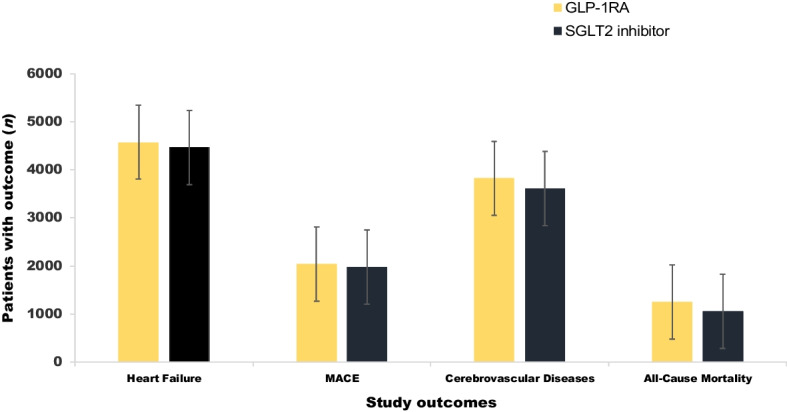
Cardiovascular outcomes and all-cause mortality in patients with NAFLD and type 2 diabetes who were new users of GLP-1RAs (*n*=38,804) or new users of SGLT2 inhibitors (*n*=38,804). MACE as a composite endpoint was defined as the first occurrence of unstable angina, myocardial infarction or revascularisation (including PCI or coronary artery bypass graft). A composite of cerebrovascular disease was defined as the first occurrence of stroke (ischaemic or haemorrhagic stroke), cerebral infarction, transient ischaemic attack, carotid intervention or surgery

Compared with the SGLT2 inhibitors group, there was no evidence that the GLP-1RAs group was associated with the following outcomes: (1) the new onset of heart failure (HR 0.97; 95% CI 0.93, 1.01); (2) a composite incidence of MACE (HR 0.95; 95% CI 0.90, 1.01); or (3) a composite of cerebrovascular diseases (HR 0.99; 95% CI 0.94, 1.03). Similarly, there was no evidence of a difference in mortality rate between the GLP-1RAs group and the SGLT2 inhibitors group (HR 1.06; 95% CI 0.97, 1.15; Fig. 2).

### GLP-1RAs vs metformin (ancillary analysis)

Several studies have shown that metformin may correct components of the metabolic syndrome [23]. Moreover, in individuals with type 2 diabetes, metformin may provide cardiovascular protection that is not solely attributed to its glucose-lowering effects, as it also has antihypertensive effects [23]. These potential cardioprotective effects may be related to the favourable actions of metformin on lipid metabolism, vascular smooth muscle, hypercoagulation, platelet hyperactivity, cardiomyocyte intracellular calcium handling and endothelial function [24]. We therefore used metformin as a control group in an analysis including 288,675 patients with NAFLD and type 2 diabetes, of which 103,667 individuals were receiving metformin (metformin group; ESM Fig 2). A well-matched population was found in the GLP-1RAs vs metformin groups (*n*=47,097 each) after PSM (ESM Figs 2 and 3). The mean follow-up was 4.8±1.1 years for the GLP-1RAs group and 4.4±1.4 years for the metformin group. ESM Tables 2 and 3 compare the baseline, demographic and clinical characteristics of the GLP-1RAs and metformin groups.

The rate of new onset of heart failure (HR 0.92; 95% CI 0.88, 0.96) and composite cerebrovascular diseases (HR 0.91; 95% CI 0.87, 0.95) was significantly lower in the GLP-1RAs group. However, the two groups had no evidence of a difference in the composite incidence of MACE (HR 0.95; 95% CI 0.90, 1.08). Mortality rate was significantly lower in the GLP-1RAs group (HR 0.62; 95% CI 0.58, 0.66; ESM Fig. 4).

### GLP-1RAs vs second- or third-line glucose-lowering medications (sensitivity analysis)

We conducted the first sensitivity analysis to compare GLP-1RAs with second- or third-line glucose-lowering medications. After PSM, the groups (*n*=52,166 each) were well matched (ESM Figs. 5 and 6). The mean follow-up was 4.8±1.1 years for the GLP-1RAs group and 4.9±1.9 years for the second- or third-line glucose-lowering medications group. A comparison of baseline demographics and clinical characteristics between GLP-1RAs and second- or third-line glucose-lowering medications is presented in ESM Tables 4 and 5.

The GLP-1RAs group had a lower rate of adverse CVEs, including new-onset heart failure (HR 0.88; 95% CI 0.85, 0.92), composite incidence MACE (HR 0.89; 95% CI 0.85, 0.94) and composite cerebrovascular events (HR 0.93; 95% CI 0.89, 0.96), compared with other second- or third-line glucose-lowering medications. Mortality rate was also significantly lower in the GLP-1RAs group (HR 0.70; 95% CI 0.66, 0.75; ESM Fig. 7).

In the second sensitivity analysis, we estimated the rates of new incidences of study outcomes by excluding patients with outcomes 2 years after the index event. The analysis led to generally consistent results, which were similar to the primary analysis between cohorts of new users of GLP-1RAs compared with new users of SGLT2 inhibitors, metformin or other second- or third-line medications. Both analyses were performed using the same methods as the primary analysis (ESM Tables 6–8).

## Discussion

In this large population-based study of individuals with NAFLD and type 2 diabetes, three major CVEs and mortality rate were compared between patients treated with and without GLP-1RAs treatment. In individuals with type 2 diabetes and NAFLD, macrovascular complications are a leading cause of morbidity and mortality [25]. Lifestyle interventions are effective for NAFLD, but adherence is challenging [26]. There is, therefore, a need for additional strategies and novel therapies. In addition to reducing liver-related complications, NAFLD management should reduce macrovascular outcomes [27]. Here, GLP-1RAs are one exciting option [28]. GLP-1RAs have been shown to improve liver health in patients with NAFLD and type 2 diabetes, as well as non-alcoholic steatohepatitis (NASH) [13, 29]. They may also reduce macrovascular outcomes. We compared GLP-1RAs with SGLT2 inhibitors and metformin and found that GLP-1RAs significantly reduced the incidence of major CVEs and mortality rate compared with metformin and second- or third-line glucose-lowering medications.

First, we compared GLP-1RAs against SGLT2 inhibitors and found no evidence of a difference in cardiovascular outcomes and mortality rate. The findings align with recent studies that showed similar results when comparing SGLT2 inhibitors and GLP-1RAs in patients with type 2 diabetes, where the NAFLD status was unknown [30]. Due to the heterogeneous nature of CVEs, sensitivity analyses were conducted to assess the robustness of our results. Several authors have demonstrated the effects of metformin on the metabolic syndrome [31, 32]. The cardioprotective effect of metformin in patients with type 2 diabetes is not only attributable to its glucose-lowering effects but also to its significant antihypertensive properties [33]. Additionally, metformin may influence lipid metabolism, smooth muscle contractions of the vascular system, hypercoagulation, platelet hyperactivity, calcium handling within cardiomyocytes and endothelial cell function [34]. Several studies have shown that metformin also lowers cholesterol levels, TG, LDL-cholesterol and HDL-cholesterol. Therefore, we used metformin as a control for the secondary analysis.

There is limited research on the head-to-head comparison of cardioprotective effects of GLP-1RAs and SGLT2 inhibitors, and the reason(s) for the similarity in the cardioprotective effect of these drug classes is not fully understood. However, some evidence suggests mechanisms involving glucose and insulin control, reducing body weight and BP, and improving blood lipid profiles [35]. GLP-1RAs improve weight loss, fatty acid metabolism and insulin signalling, which may benefit cardiovascular health [36]. Similarly, SGLT2 inhibitors have been shown to improve insulin sensitivity and lower blood sugar levels, which can also reduce the risk of CVEs [37]. GLP-1RAs and SGLT2 inhibitors also have pleiotropic effects, including blood glucose-dependent and -independent mechanisms, effects on the renin–angiotensin–aldosterone system and favourable effects on left ventricular and renal functions [35, 36]. A study by the UK Prospective Diabetes Study Group has shown that long-term intensive glucose control could reduce mortality rate, possibly due to the potential benefit of these medications [37]. Comparison of the cardiovascular outcomes of GLP-1RAs and SGLT2 inhibitors may help the clinician to select the best personalised treatment approach for patients with type 2 diabetes [38]. Further research is needed to fully understand the similarities and differences in the cardioprotective effects of GLP-1RAs and SGLT2 inhibitors.

Interestingly, GLP-1RAs showed significantly more reduction in cardiovascular endpoints than metformin. In particular, mortality rate was reduced in the GLP-1RAs group compared with the metformin group. This result contrasts a recent study that found no evidence of differences in macrovascular disease prevention for GLP-1RAs compared with metformin in individuals with type 2 diabetes (whose hepatic steatosis status was unclear) [39]. We hypothesise that a few mechanisms may have contributed to the observed cardioprotective effects of GLP-1RAs in our study: (1) this class of glucose-lowering drugs induces weight loss, which has a positive impact and results in a better cardioprotective effect; (2) these drugs may increase heart rate variability, which is associated with a lower rate of adverse CVEs; and (3) GLP-1RAs improve insulin sensitivity and reduce BP, which may reduce the risk of CVEs. However, future studies are needed to explore the pathophysiology of GLP-1RAs in individuals with type 2 diabetes and NAFLD and to determine the optimal use of GLP-1RAs to prevent adverse CVEs.

To minimise confounding effects, we compared GLP-1RAs with second- or third-line glucose-lowering medications. By adjusting for microvascular complications, we could explore the impact of including a reference group at the same disease stage. GLP-1RAs showed significantly more reduction of cardiovascular endpoints than other second- or third-line glucose-lowering medications. In particular, mortality rate was reduced in the GLP-1RAs group compared with the second- or third-line medications. Patients who received GLP-1RAs were also associated with a significantly lower incidence of CVEs than individuals in the metformin and the second- or third-line glucose-lowering medications groups. In particular, the incidence of MACE was significantly lower in the GLP-1RAs group. Compared with individuals in the metformin and second- or third-line glucose-lowering medications groups, major CVEs were significantly lower in individuals with NAFLD and type 2 diabetes who received GLP-1RAs but not different from those who received SGLT2 inhibitors. The study results align with current guidelines, which prefer GLP-1RAs over second- or third-line glucose-lowering medications in patients with type 2 diabetes [40, 41].

Patients and providers should be made aware of adverse CVEs in future studies, and pharmacologic and non-pharmacologic approaches should be examined to reduce NAFLD and adverse CVEs in individuals with type 2 diabetes. Long-term adverse CVEs in individuals with NAFLD and type 2 diabetes should also be examined prospectively. To reduce the development of adverse CVEs among patients with NAFLD and type 2 diabetes, it is also essential to have routine cardiovascular risk assessments and timely implementation of GLP-1RAs and SGLT2 inhibitors.

### Strengths and limitations

Our study had several strengths. First, we analysed large population-based, multicentre data collected nationwide from healthcare organisations. Second, baselines and potential confounders were adjusted to create a robust control group. Third, the large sample size in the propensity-matched analyses resulted in narrower CIs, demonstrating higher precision. Hence, using PSM provided additional strength to the study results. Furthermore, we used a new user cohort study design as a comparator, which reduced the potential for unmeasured confounding [42]. Lastly, we ensured that the sample comprised only individuals with NAFLD and type 2 diabetes using an exhaustive list of quality checks and diagnosis codes, including but not limited to the exclusion of alcohol-related disorders, alcohol abuse, alcohol dependence and alcohol-related liver diseases. We believe that this is the largest published study comparing adverse CVEs in patients with NAFLD and type 2 diabetes with and without GLP-1RA treatment.

Our study also had some limitations. First, the retrospective design and the reliance on an EHR-based database limited our results. Whenever patient information is translated into diagnosis codes, data from EHR-based databases are susceptible to errors in coding. Standardised measures were used to identify cases to minimise documentation errors. Second, we did not account for some residual confounding even after adjusting potential confounders in our analyses. However, we used new users as a cohort to reduce the potential for unmeasured confounding. Additionally, we did not grade comorbid conditions at baseline, which might have caused some selection bias in the cohorts. Third, we did not include imaging modalities to confirm the NAFLD diagnosis (e.g. ultrasonography, computed tomography, MRI or elastography), as it was not available in our dataset. In future studies, it would be ideal to link NASH and type 2 diabetes separately to adverse CVEs incidence and mortality rate. We acknowledge that there were some differences in the comparison between GLP-1RAs and other glucose-lowering agents, particularly concerning HbA_1c_ levels, the use of insulin and the use of SGLT2 inhibitors, which may have influenced the results. Excluding patients with prior CVD and cerebrovascular disease could have influenced the outcomes of our study and potentially impacted the comparison between GLP-1RAs and SGLT2 inhibitors. Finally, the inclusion criteria were not based on histological diagnosis, which could lead to some data contamination due to misdiagnosis.

### Conclusions

The results of this large population-based cohort study indicate that in patients with NAFLD and type 2 diabetes, GLP-1RAs and SGLT2 inhibitors are associated with lower incidences of macrovascular diseases and all-cause mortality compared with other glucose-lowering drugs. Though our study provides novel information, randomised controlled trials and additional observational studies are needed to corroborate our findings, given the significant morbidity and mortality of CVEs in patients with NAFLD and type 2 diabetes. Compared with metformin and second- or third-line glucose-lowering medication groups, major adverse CVEs were significantly lower in patients with NAFLD and type 2 diabetes taking GLP-1RAs, but not different from those taking SGLT2 inhibitors. Routine cardiovascular risk assessments and timely implementation of GLP-1RAs and SGLT2 inhibitors are also essential for reducing the adverse CVEs in patients with NAFLD and type 2 diabetes.

### Supplementary Information

Below is the link to the electronic supplementary material.

Supplementary file1 (PDF 840 KB)

## Data Availability

No additional data are available.

## Abbreviations

CVE: Cardiovascular event
EHR: Electronic health record
GLP-1RA: Glucagon-like peptide-1 receptor agonist
MACE: Major adverse cardiovascular events
NAFLD: Non-alcoholic fatty liver disease
NASH: Non-alcoholic steatohepatitis
PCI: Percutaneous coronary intervention
PSM: Propensity score matching
SGLT2: Sodium–glucose cotransporter 2
SMD: Standardised mean difference
TG: Triacylglycerol
