# Supplementary material for: Cardiovascular and mortality outcomes with GLP-1 receptor agonists vs other glucose-lowering drugs in individuals with NAFLD and type 2 diabetes: a large population-based matched cohort study
Source: Diabetologia. 2023 Dec 20;67(3):483–93. doi: 10.1007/s00125-023-06057-5 (PMC10844347; doi:10.1007/s00125-023-06057-5)
Supplement: Supplementary file 1 — Supplementary file1 (PDF 840 KB) [file 125_2023_6057_MOESM1_ESM.pdf]

Electronic Supplementary Material (ESM) Online Content

eAppendix. Supplementary Methods

ESM Fig 1. Propensity score density graph for the users of glucagon-like peptide-1 receptor agonists versus sodium-glucose cotransporter-2 inhibitors users among patients with nonalcoholic fatty liver disease and type 2 diabetes before and after propensity score matching.

ESM Fig 2: Study Flow Chart of Patient Selection in the Study Cohort for New Users of Glucagon-Like Peptide-1 Receptor Agonist and New Users of Metformin (Active-Comparator).

ESM Fig 3: Propensity score density graph for the glucagon-like peptide-1 receptor agonists users versus metformin users among patients with nonalcoholic fatty liver disease and type 2 diabetes before and after propensity score matching.

ESM Fig 4: Cardiovascular outcomes and all-cause mortality between the new users of GLP-1RAs vs. metformin in patients with nonalcoholic fatty liver disease and type 2 diabetes mellitus.

ESM Fig 5: Study Flow Chart of Patient Selection in the Study Cohort for New Users of Glucagon-Like Peptide-1 Receptor Agonist and New Users of second- or third-line glucose-lowering drugs (Active-Comparator).

ESM Fig 6: Propensity score density graph for the users of glucagon-like peptide-1 receptor agonists versus the second- or third-line glucose-lowering medication users among patients with nonalcoholic fatty liver disease and type 2 diabetes before and after propensity score matching.

ESM Fig 7: Cardiovascular outcomes and all-cause mortality between the new users of GLP-1RAs vs. second-or third-line glucose-lowering drugs in patients with nonalcoholic fatty liver disease and type 2 diabetes mellitus.

ESM Table 1: Laboratory findings of new GLP-1A users vs. matched new SGLT-2i users in patients with NAFLD and type 2 diabetes.

ESM Table 2: Baseline characteristics of new GLP-1A users vs matched new metformin users in patients with NAFLD and type 2 diabetes.

ESM Table 3: Laboratory findings of new GLP-1A users vs matched new metformin users in patients with NAFLD and type 2 diabetes.

ESM Table 4: Baseline characteristics of new GLP-1RA users vs. matched new Second- or-third-line glucose-lowering users in patients with NAFLD and type 2 diabetes.

ESM Table 5: Laboratory findings of new GLP-1RA users vs. matched new Second -or third-line glucose-lowering drug users in patients with NAFLD and type 2 diabetes.

ESM Table 7: Sensitivity analysis to assess the cardiovascular outcomes and all causes of mortality between the new users GLP-1RA vs. Metformin in patients with NAFLD and type 2 diabetes after excluding index events within 2 years after the index date.

ESM Table 8: Sensitivity analysis to assess the cardiovascular outcomes and all causes of mortality between the new users GLP-1RA vs. Second- or third-line glucose-lowering medications in patients with NAFLD and type 2 diabetes after excluding index events within 2 years after the index date.

eAppendix. Supplementary Methods

Data Source

We used TriNetX (Cambridge, MA, USA), a global federated health research network providing real-time access to electronic health records (EHRs). TriNetX platform de-identifies and aggregates EHR data from 66 healthcare organizations (HCOs), most of which are large academic medical institutions with both inpatient and outpatient facilities at multiple locations across 50 states in the United States. The Real-time access to health insurance portability and accountability act– de-identified, compliant, and longitudinal clinical data to member HCOs is provided cloud-based. The de-identified clinical data, such as diagnoses, procedures, medications, laboratory values, and genomic information, are continuously aggregated directly from the EHR of the participating HCOs. Participating HCOs include outpatient, inpatient, and specialty care services and provide care to a diverse patient population from different ethnicity, age groups, geographical region, and income levels. Both the patients and HCOs, as data sources, stay anonymous.

As a federated network, TriNetX data have been granted a waiver from the Western institutional review board since only aggregated counts and statistical summaries of de-identified information without any protected health information were received from participating HCOs. In addition, no study-specific activities are performed in retrospective analyses.

Standardizing the terminology and data quality check:

The TriNetX software verifies the basic formatting to confirm that data are appropriately characterized. Patient counts were rounded up to the nearest 10 in our analysis to safeguard protected health information. TriNetX has production capabilities that have been tested that map data extensively from each of these structures to the standard model within TriNetX and can extract details of interest from the narrative content of clinical documents using natural language processing. The contributing EHR systems used United Medical Language System (UMLS) for coding. TriNetX maps the data to a standard and controlled set of clinical terminologies, for example, mapping disease terms from Systematized Nomenclature of Medicine—Clinical Terms (SNOMED CT) to International Classification of Diseases, and Clinical Modification (ICD-9 and 10 CM), drug terms from National Drug Codes (NDCs) to RxNorm. TriNetX enforces a list of required fields (e.g., patient identifier) and rejects those records where the required data is lacking. Referential integrity checking confirms that data spanning multiple database tables can be successfully joined together. TriNetX requires at least 1 non-demographic fact for a patient to be calculated in a given data set. Patient records with only demographic information are not included in data sets. As the data are refreshed, the TriNetX software monitors change in data volumes over time to ensure data validity.

Selection of Patients:

The search was conducted following the criteria provided by TriNetX to identify potential patients. These codes included the ICD-9, 10 CM. We combined patients into a single cohort of

NAFLD/NASH defined by a diagnosis of NAFLD based on (571.8, K76.0, and K75.81). 571.5 , K74.6 Other and unspecified cirrhosis othe f liver.

Exclusion criteria:

We excluded the patients if they had a diagnosis of another defined cause of liver disease other than NAFLD, including acute alcohol abuse or chronic alcohol abuse, alcoholic liver disease, toxic liver disease, viral hepatitis, Wilson's disease, autoimmune hepatitis, Gaucher disease, primary biliary cholangitis, hemochromatosis, primary sclerosing cholangitis. Diagnosis codes based on the exclusion criteria are listed below. 070, Viral hepatitis; 571.6, 576.1, Autoimmune liver disease (AIH, PBC, PSC); 275.0, Hemochromatosis; 275.1, Wilson's disease; 277.6, Alpha-1-antitrypsin deficiency; 453.0, Budd-Chiari syndrome; 571.4, Chronic hepatitis, unspecified; 571.6, Secondary or unspecified biliary cirrhosis, 571.8, nonalcoholic fatty liver disease; 303, 305.0, alcohol use disorder; 291, 357.5, 425.5, 535.3, 980.1, 980.9 somatic consequences of alcohol (except ALD); 305.1-9, drug use disorders except nicotine/caffeine; 571.5, Cirrhosis, compensated; 456.1, 456.21, Esophageal varices, not bleeding; 456.0, 456.20, Esophageal varices, bleeding; 789.5, Ascites; 572.2, hepatic encephalopathy; 572.4, Hepatorenal syndrome; 572.3, Portal hypertension; V427, Liver transplantation status, 155.0, hepatocellular carcinoma; 155.2, Liver cancer, unspecified; 572.8, Chronic or unspecified liver failure; 570, Acute or subacute liver failure; 571,9, Hepatic fibrosis or sclerosis or fibrosis with sclerosis. K72 Acute and subacute hepatic failure without coma necrosis; K76.2 Central hemorrhagic necrosis of liver necrosis; K700 Alcoholic fatty liver hepatitis; K7010 Alcoholic hepatitis without ascites hepatitis; K7030 Alcoholic cirrhosis. Of liver without ascites cirrhosis. K709 Alcoholic liver disease, unspecified cirrhosis; K730 Chronic persistent hepatitis, not elsewhere classified hepatitis; K732 Chronic active hepatitis, not elsewhere classified hepatitis; K738 Other chronic hepatitis, not elsewhere classified hepatitis; K739 Chronic hepatitis, unspecified hepatitis; K740 Hepatic fibrosis; K74.1 Hepatic sclerosis cirrhosis; K743 Primary biliary cirrhosis;K74.4 Secondary biliary cirrhosis;K74.5 Biliary cirrhosis, unspecified cirrhosis;;K75.4 Autoimmune hepatitis;K76.7 Hepatorenal syndrome cirrhosis;K77 Liver disorders in diseases classified elsewhere hepatitis;K71.6 Toxic liver disease with hepatitis, not elsewhere classified hepatitis;B17.0 Acute delta-(super) infection of hepatitis B carrier hepatitis;B1710 Acute hepatitis C without hepatic coma hepatitis;B17.2 Acute hepatitis E hepatitis;B17.8 Other specified acute viral hepatitis;B18.2 Chronic viral hepatitis C hepatitis;B18.8 Other chronic viral hepatitis;B18.9 Chronic viral hepatitis, unspecified hepatitis;B0081 Herpesviral hepatitis hepatitis;B15.0 Hepatitis A with hepatic coma

hepatitis;B15.9 Hepatitis A without hepatic coma hepatitis;B16.0 Acute hepatitis B with delta-agent with hepatic coma hepatitis;B16.1 Acute hepatitis B with delta-agent without hepatic coma hepatitis;B16.2 Acute hepatitis B without delta-agent with hepatic coma hepatitis;B16.9 Acute hepatitis B without delta-agent and without hepatic coma hepatitis;B17.11 Acute hepatitis C with hepatic coma hepatitis;B17.2 Acute hepatitis E hepatitis;B17.8 Other specified acute viral hepatitis;B179 Acute viral hepatitis, unspecified hepatitis;B18.0 Chronic viral hepatitis B with delta-agent hepatitis;B18.1 Chronic viral hepatitis B without delta-agent hepatitis;B18.2 Chronic viral hepatitis C hepatitis;B19.0 Unspecified viral hepatitis with hepatic coma hepatitis;B19.10 Unspecified viral hepatitis B without hepatic coma hepatitis;B19.11 Unspecified viral hepatitis B with hepatic coma hepatitis; B19.20 Unspecified viral hepatitis C without hepatic coma hepatitis; B19.21 Unspecified viral hepatitis C with hepatic coma hepatitis; B19.9 Unspecified viral hepatitis without hepatic coma hepatitis; B25.1 Cytomegaloviral hepatitis; B25.1 Cytomegaloviral hepatitis; B26.81 Mumps hepatitis; B58.1 Toxoplasma hepatitis; B942 Sequelae of viral hepatitis; K70.11 Alcoholic hepatitis with ascites hepatitis; E83.01, Wilson's disease; K76.9 Liver disease, unspecified hepatitis; K72.90 Hepatic failure, unspecified without coma cirrhosis; K73.1 Chronic lobular hepatitis, not elsewhere classified hepatitis; K75.2 Nonspecific reactive hepatitis; B20, human immunodeficiency virus; K75.3 Granulomatous hepatitis, not elsewhere classified hepatitis; K75.89 Other specified inflammatory liver diseases hepatitis; K70.31 Alcoholic cirrhosis of liver with ascites cirrhosis; F10.2 Alcohol dependence; K71.7 Toxic liver disease with fibrosis and cirrhosis of the liver.

Baseline characteristics related codes:

Hypertension, E11.1, Type 2 diabetes; I60-I69 Cerebrovascular diseases; K21, Gastroesophageal reflux disease; J40-J47, Chronic respiratory diseases; G47.33 Obstructive sleep apnea; N18 Chronic kidney diseases; M81 Osteoporosis; E11.21 Nephropathy; E11.31 Retinopathy; E11.42 Polyneuropathy; N00-N08 Glomerular diseases.

Diagnosis and procedure codes to assist in identifying cardiovascular conditions:

Coronary artery disease: I25, I259; Acute myocardial infarction: ICD: i21.X and i21.XX, i22.X and i22.XX, i23.X and i23.XX; Stroke: i63.X and i63.XX and i63.XXX; Heart failure: I50.X; I50.XX; I50. XXX; Cerebrovascular event: Diagnoses and procedure: 433·X1, 434·X1, 436·0, 430·X, 431·X, I67.XX, I60.X; 38·12, 0·61, 0·63, CPT: 37215, 37216, 0075T, 0076T, 35301, 37205, 37206; Coronary artery disease: ICD for diagnose and procedure: 410.X, 411.X, 411.X AND 414.X, 36.01, 36.02, 36.03, 36.05, 36.06, 36.07, 36.10, 36.11,

36.12, 36.13, 36.14, 36.15, 36.16, 36.17, 36.19, 36.31, 36.32, 36.33, 36.64; CPT: 92982, 92984, 92995, 92996, 92980, 92981, 33510, 33511,33512, 33513, 33514, 33516, 33517, 33518, 33519, 33521, 33522, 33523, 33530, 33533, 33534, 33535, 33536, 93539, 9354.

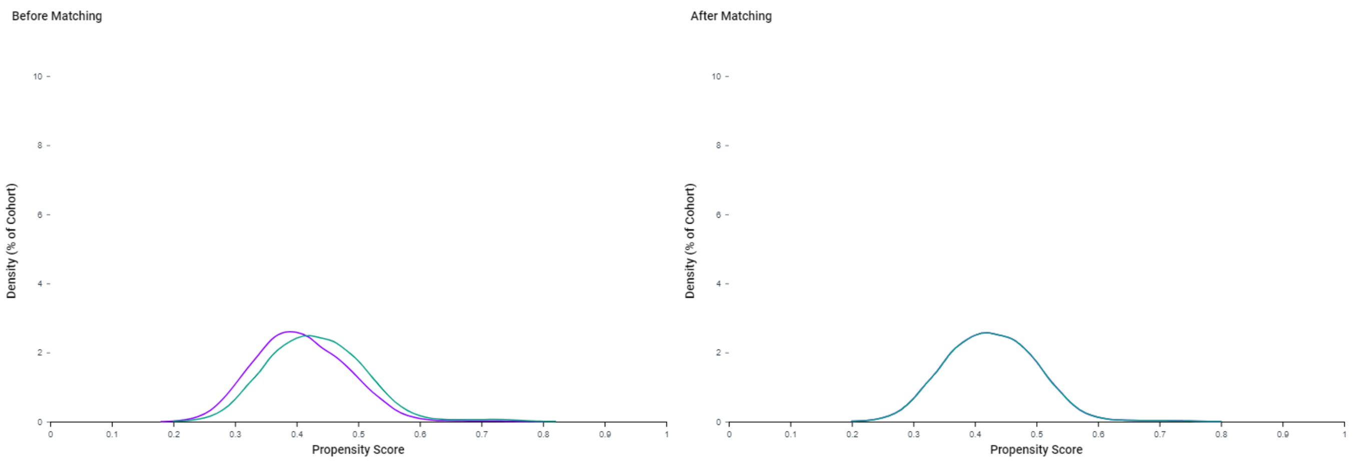

ESM Fig 1: Propensity score density graph for the users of glucagon-like peptide-1 receptor agonists (Purple line) versus sodium-glucose cotransporter-2 inhibitors (Green line) users among patients with nonalcoholic fatty liver disease and type 2 diabetes before and after propensity score matching.

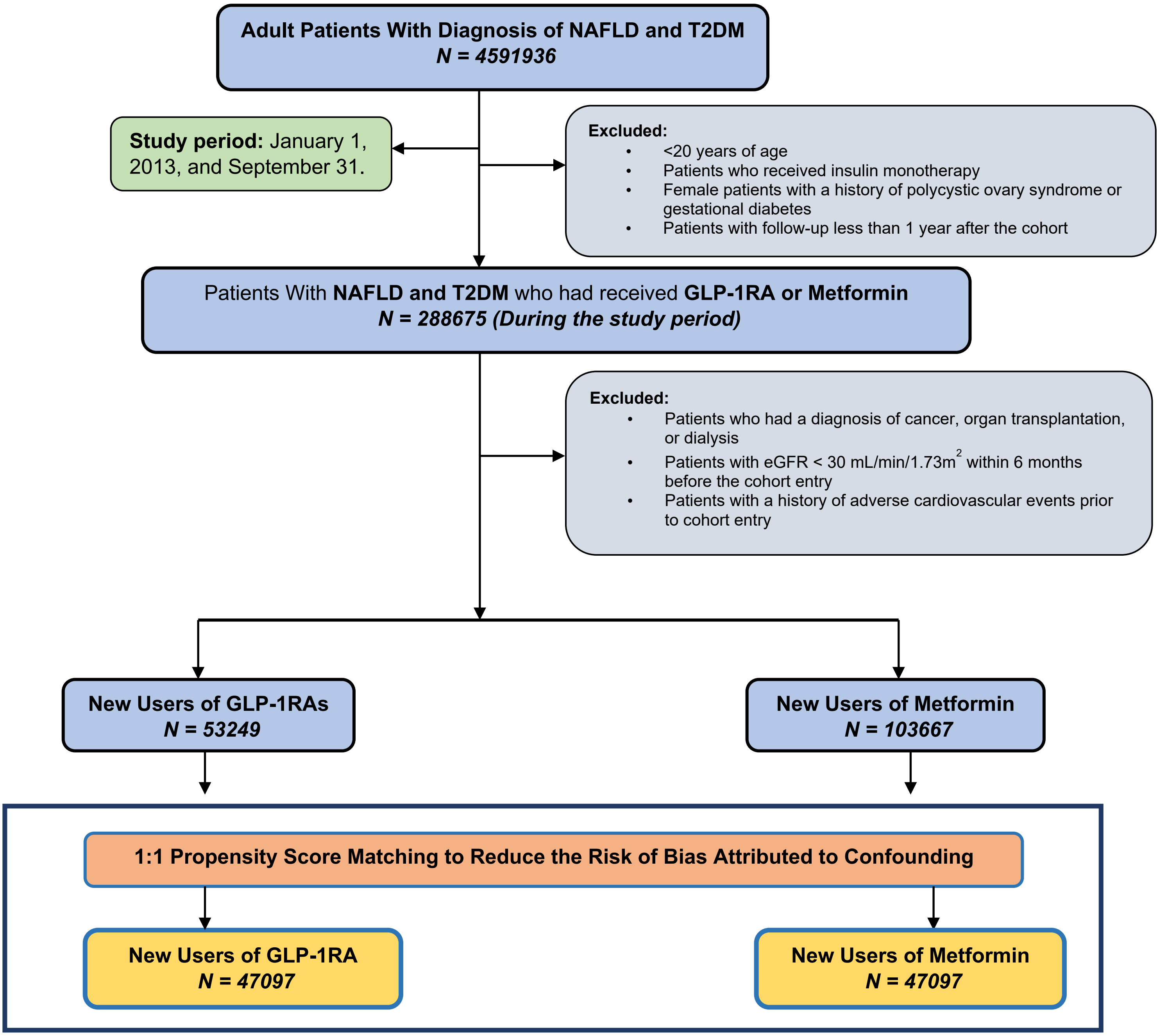

ESM Fig 2: Study Flow Chart of Patient Selection in the Study Cohort for New Users of Glucagon-Like Peptide-1 Receptor Agonist and New Users of Metformin (Active-Comparator). NAFLD, nonalcoholic fatty liver disease; T2DM, type 2 diabetes mellitus ;GLP-1RA, glucagon-like peptide-1 receptor agonists; eGFR, estimated glomerular filtration rate.

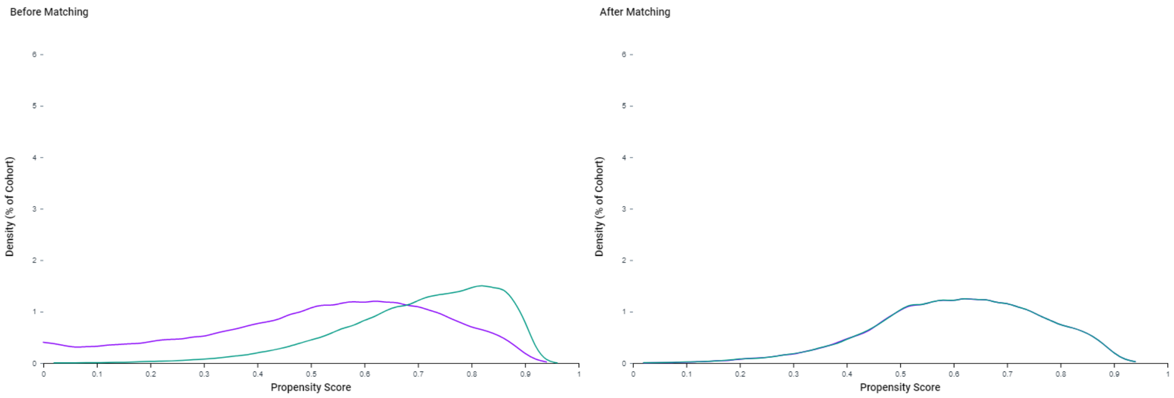

ESM Fig 3: Propensity score density graph for the users of glucagon-like peptide-1 receptor agonists (Purple line) versus metformin (Green line) users among patients with nonalcoholic fatty liver disease and type 2 diabetes before and after propensity score matching.

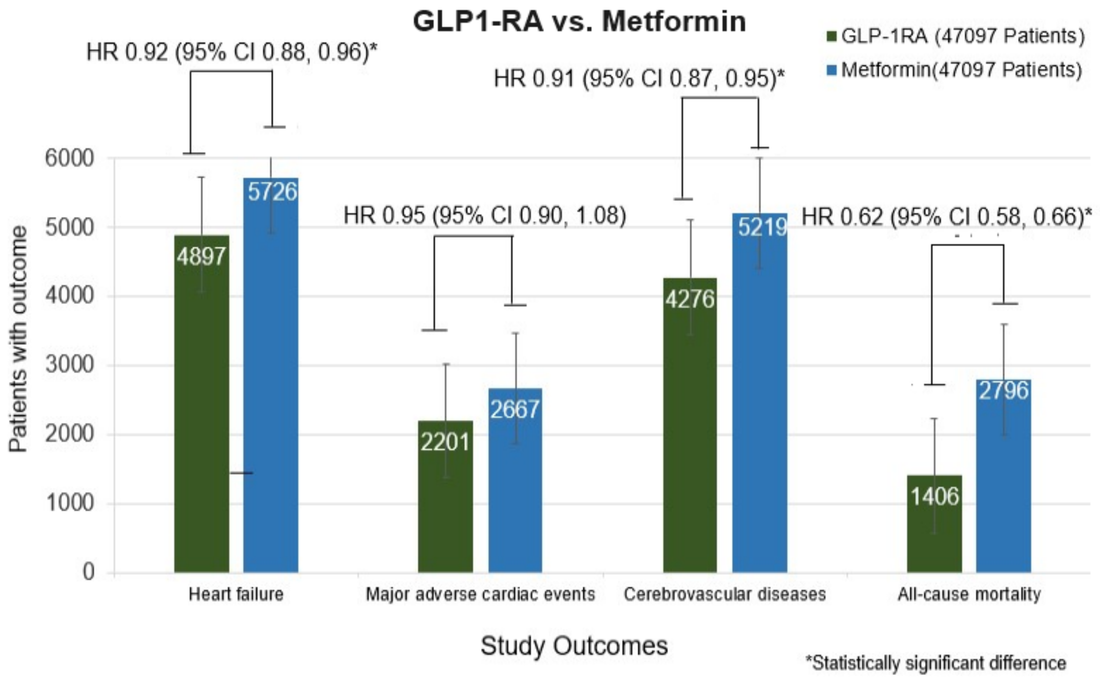

ESM Fig 4: Cardiovascular outcomes and all-cause mortality between the new users of GLP-1RAs vs. metformin in patients with nonalcoholic fatty liver disease and type 2 diabetes mellitus. GLP-1RA, glucagon-like peptide-1 receptor agonists; Major adverse cardiac events as a composite endpoint was defined as the first occurrence of unstable angina, or myocardial infarction, and revascularization, including percutaneous coronary intervention or coronary artery bypass graft. A composite of cerebrovascular disease was defined as the first occurrence of stroke (ischemic or hemorrhagic stroke), cerebral infarction, transient ischemic attack, carotid intervention, or surgery.

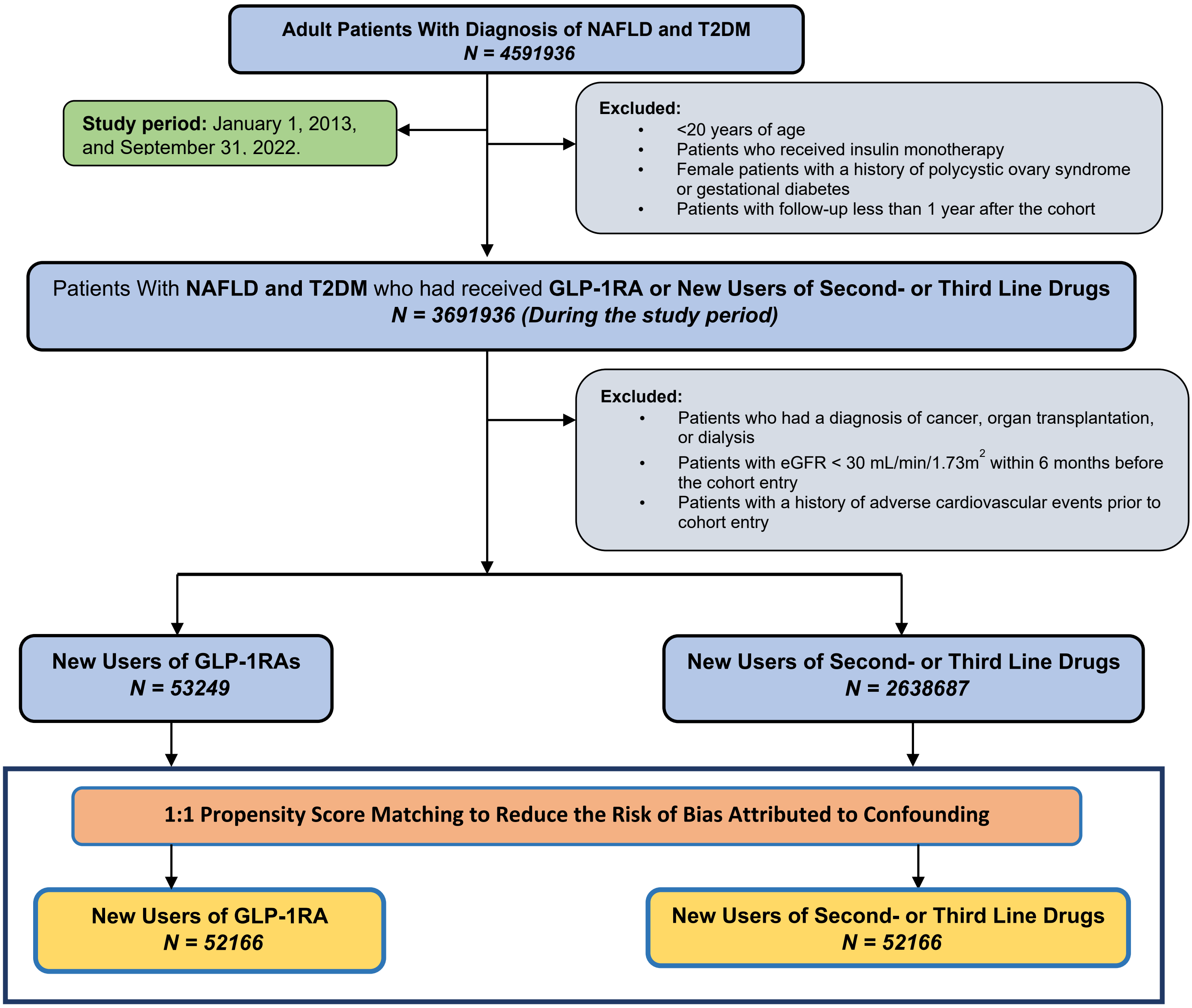

ESM Fig 5: Study Flow Chart of Patient Selection in the Study Cohort for New Users of Glucagon-Like Peptide-1 Receptor Agonist and New Users of second- or third-line glucose-lowering medication (Active-Comparator). NAFLD, nonalcoholic fatty liver disease; T2DM, type 2 diabetes mellitus ;GLP-1RA, glucagon-like peptide-1 receptor agonists; eGFR, estimated glomerular filtration rate.

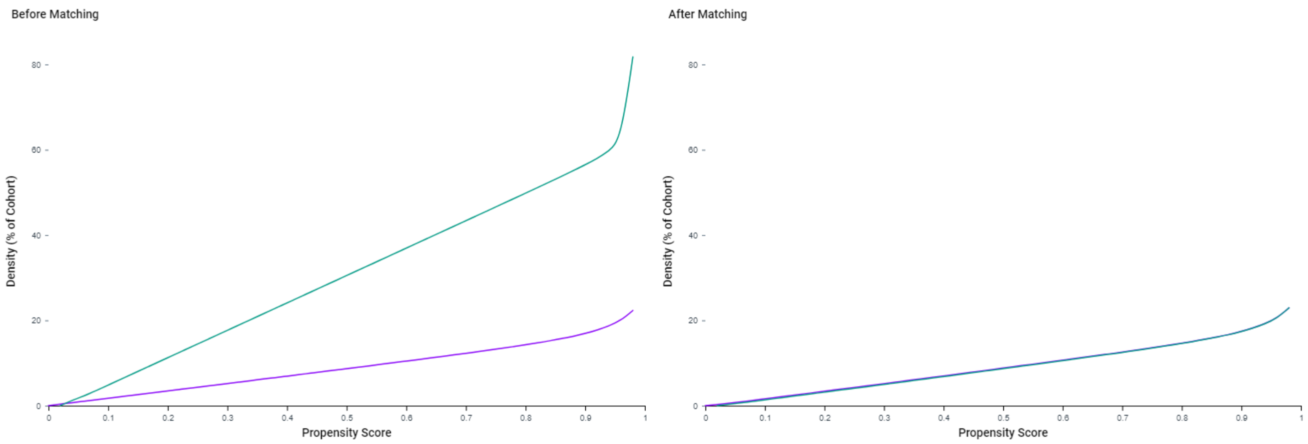

ESM Fig 6: Propensity score density graph for the users of glucagon-like peptide-1 receptor agonists (Purple line) versus the second- or third line glucose-lowering medication (Green line) users among patients with nonalcoholic fatty liver disease and type 2 diabetes before and after propensity score matching.

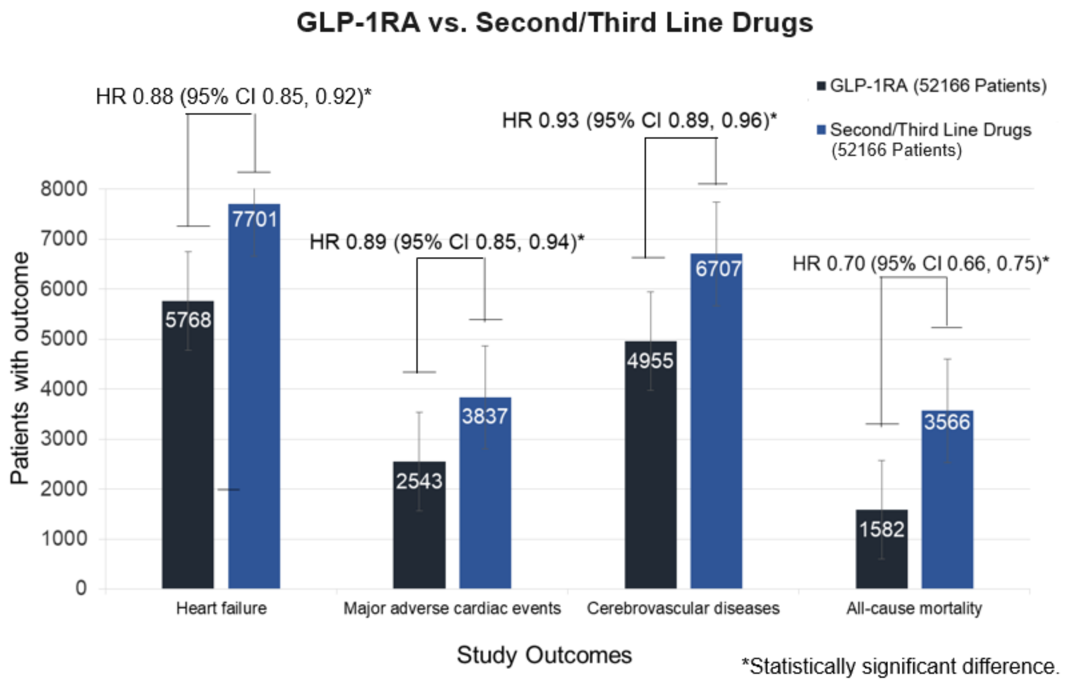

ESM Fig 7: Cardiovascular outcomes and all-cause mortality between the new users of GLP-1RAs vs. second-or third line antidiabetic drugs in patients with nonalcoholic fatty liver disease and type 2 diabetes mellitus. GLP-1RA, glucagon-like peptide-1 receptor agonists; Major adverse cardiac events as a composite endpoint was defined as the first occurrence of unstable angina, or myocardial infarction, and revascularization, including percutaneous coronary intervention or coronary artery bypass graft. A composite of cerebrovascular disease was defined as the first occurrence of stroke (ischemic or hemorrhagic stroke), cerebral infarction, transient ischemic attack, carotid intervention, or surgery.

ESM Table 1: Laboratory findings of new GLP-1A users vs matched new SGLT-2i users in patients with NAFLD and type 2 diabetes

Variables	Before propensity matching			After propensity matching		
	**GLP-1RA** (N=53249)	**SGLT-2i** (N=39795)	**SMD**	**GLP-1RA** (N=38804)	**SGLT-2i** (N=38804)	**SMD**
**Vital signs:** *mean ± SD*						
SBP, mmHg	130 ± 17.2	130 ± 17.3	0.0159	131 ± 17.4	130 ± 17.2	0.0172
DBP ,mmHg	77.1 ± 11	76.8 ± 10.9	0.0278	76.9 ± 10.9	76.9 ± 10.9	0.0034
**Liver function test:** *mean ± SD*						
ALT (U/L)	43.1 ± 44.6	43.5 ± 48.7	0.0090	43.6 ± 47.7	43.6 ± 49	0.0003
AST (U/L)	34.4 ± 28.6	34.7 ± 32.2	0.0100	34.5 ± 29	34.8 ± 32.3	0.0079
T.Bil (µmol/L)	9.82± 11.51	10.50 ± 11.42	0.0518	10.16 ± 13.08	10.45 ± 14.54	0.0208
ALP (U/L)	90.1 ± 41.6	90.2 ± 45.8	0.0041	89.4 ± 41.2	90.2 ± 45.3	0.0193
Serum Albumin (g/L)	40.80 ± 4.91	40.10 ±4.97	0.0505	40.90 ± 4.91	40.10 ± 4.95	0.0322
**Coagulation profile:** *mean ± SD*						
PT (sec)	12.50 ± 6.20	12.70 ± 6.14	0.0240	12.60 ± 6.38	12.70 ± 6.17	0.0060
INR	1.20 ± 1.90	1.19 ± 1.70	0.0035	1.21 ± 1.87	1.19 ± 1.73	0.0079
APTT (sec)	30.80 ± 11.60	31.30 ± 11.60	0.0423	30.90 ± 11.50	31.20 ± 11.60	0.0260
**Complete blood count:** *mean ± SD*						
Hemoglobin (g/L),	135 ± 18	137 ± 18.5	0.1007	136 ± 18.2	137 ± 18.4	0.0328
Platelets (10^9/L)	247 ± 82.30	240 ± 82.70	0.0917	242 ± 82.10	240 ± 82.60	0.0184
HCT, (Proportion of 1.0)	0.40± 0.07	0.41 ± 0.07	0.0810	0.41 ± 0.07	0.41 ± 0.07	0.0356
**Metabolic profile:** *mean ± SD*						
HbA1C (Proportion of total hemoglobin)	0.08± 0.02	0.08± 0.02	0.0022	0.08± 0.02	0.08± 0.02	0.0020
Fasting blood glucose, (mmol/L)	8.38±3.45	8.71±3.45	0.0919	8.49±3.40	8.71±3.46	0.0614
Creatinine, (µmol/L)	77.78± 291.27	74.65± 222.65	0.0133	79.30± 280.60	74.42± 225.70	0.0192
GFR (mL/min/1.73 m²)	83.9 ± 28	84.1 ± 27.8	0.0046	83 ± 27.4	84.3 ± 27.8	0.0482
Total protein (g/L)	70.90 ± 10.10	70.10 ± 10.90	0.0127	70.90 ± 1.80	70.10 ± 10.90	0.0091
BUN, (mmol/L)	5.57 ± 2.86	5.68 ± 2.74	0.0398	5.75 ± 2.88	5.64 ± 2.69	0.0287
**Cardiac markers,** *mean ± SD*						
LDH, (µkat/L)	4.16 ± 5.66	4.28 ± 5.39	0.0220	4.23 ± 6.55	4.23 ± 5.16	< 0.0001
**Inflammatory markers,** *mean ± SD*						
Ferritin (µg/L)	176 ± 334	215 ± 687	0.0727	186 ± 351	214 ± 686	0.0509
CRP (ug/L)	1.89 ± 3.97	1.94 ± 4.06	0.0123	1.92 ± 4.01	1.92 ± 4.01	< 0.0001
Urate (mmol/L)	352.12 ± 129.67	366.29 ± 143.94	0.0328	353.91± 136.21	353.91 ± 143.94	0.0026
ESR(mm/h)	26.6 ± 22.8	25.7 ± 22.9	0.0419	25.7 ± 22.7	25.6 ± 22.8	0.0034
**Lipid profile:** *mean ± SD*						
CH (mmol/L)	4.43 ± 1.26	4.38± 1.34	0.0508	4.38 ± 1.26	4.38± 1.34	0.0061
TG (mmol/L)	2.41 ± 2.66	2.50 ± 2.79	0.0353	2.44 ± 2.84	2.51 ± 2.81	0.0240
LDL (mmol/L)	2.38± 0.98	2.29 ± 1.00	0.0794	2.31 ± 0.97	2.30 ± 1.00	0.0111
HDL (mmol/L)	1.06 ± 0.35	1.04 ± 0.36	0.0648	1.05 ± 0.35	1.04 ± 0.36	0.0181

Abbreviations: NAFLD, nonalcoholic fatty liver disease; GLP-1RA, glucagon-like peptide-1 receptor agonists; SMD, standard mean difference; SD, standard deviation; SBP, systolic blood pressure; DBP, diastolic blood pressure; ALT, alanine transaminase ; AST, aspartate aminotransferase; T.Bil, total bilirubin;ALP, alkaline phosphatase; PT, prothrombin time; INR, international normalized ratio; APTT, activated partial thromboplastin; HCT, hematocrit; HbA1C, hemoglobin A1C ; BUN, blood urea nitrogen; GFR, glomerular filtration rate ; LDH, lactate dehydrogenase; CRP, ; CH, cholesterol; TG, triglycerides; LDL, low-density lipoprotein ; HDL, high-density lipoprotein.

ESM Table 2: Baseline characteristics of new GLP-1A users vs matched new metformin users in patients with NAFLD and type 2 diabetes

Variables	Before propensity score match			After propensity score match		
	GLP-1A (N=53249)	**Metformin** (N=103667)	**SMD**	GLP-1A (N=47097)	**Metformin** (N=47097)	**SMD**
**Age in years,** mean±SD	54.6 ± 12	55.6 ± 13.7	0.0741	54.7 ± 12	54.9 ± 13.7	0.0153
**Sex, n (%),** *Female*	32188(60.4)	56066(54.0)	0.1289	27860(59.1)	28196(59.8)	0.0145
**Ethnicity, n (%)**						
Hispanic or Latino	5161(9.6)	13410(12.9)	0.1025	4679(9.9)	4125(8.7)	0.0404
**Race, n (%)**						
White	38990 (73.2)	71511(68.9)	0.0937	34323(72.8)	34907(74.1)	0.0281
Black or African Americans	7649(14.3)	14670(14.1)	0.0061	6640(14.0)	6370(13.5)	0.0166
Others	5301 (9.9)	13481(13.0)	0.0958	4907(10.4)	4683(9.9)	0.0157
**Nicotine dependence**, n(%)	9492(17.8)	17785(17.1)	0.0176	8317(17.6)	8068(17.1)	0.0139
**BMI (kg/m^2),** mean±SD	37.1±6.85	34.9 ±7.12	0.3106	37 ± 6.82	35.6 ± 7.12	0.2042
**Comorbidities,** n (%)						
Hypertension	40674(76.3)	64941(62.6)	0.3019	34880(74.0)	34718 (73.7)	0.0078
Hyperlipidemia	32759(61.5)	46412 (44.7)	0.3405	27426(58.2)	27386 (58.1)	0.0017
Chronic lower respiratory diseases	17894(33.6)	26714 (25.7)	0.1721	14962(31.7)	14806 (31.4)	0.0071
Hypercholesterolemia	12968(24.3)	17942 (17.3)	0.1742	10876(23.0)	10374 (22.0)	0.0255
Diabetic polyneuropathy	9289(17.4)	6769 (6.5)	0.3409	5835(12.3)	5724 (12.1)	0.0072
Chronic kidney disease	7534(14.1)	8179 (7.8)	0.2009	5657(12.0)	5472 (11.6)	0.0122
Peripheral vascular diseases	4094(7.6)	5379 (5.1)	0.1020	3206(6.8)	3092 (6.5)	0.0097
Diabetic retinopathy	3629(6.8)	2785 (2.6)	0.1950	2277(4.8)	2163 (4.5)	0.0114
Diabetic nephropathy	3795(7.1)	2248 (2.1)	0.2372	2112(4.4)	1971 (4.1)	0.0147
Glomerular diseases	1841(3.4)	1537 (1.4)	0.1275	1115(2.3)	1166(2.4)	0.0070
cirrhosis of liver	4017(7.5)	6624 (6.3)	0.0453	3538(7.5)	2890 (6.1)	0.0546
Osteoporosis	2514(4.7)	4366 (4.2)	0.0247	2081(4.4)	2411(5.1)	0.0329
Sleep apnea	18993(35.6)	21449(20.6)	0.3355	14740(31.2)	14813(31.4)	0.0032
**Cardiovascular medications,** n (%)						
Beta-blockers	23660(44.4)	36378(35.0)	0.1918	20017(42.5)	19667(41.7)	0.0151
Antiarrhythmics	25198(47.3)	35236(33.9)	0.2739	21298(45.2)	19156(40.6)	0.0920
Antilipemic agents	36367(68.2)	50018(48.2)	0.4152	30,618(65.0)	30641(65.0)	0.0010
ACE inhibitors	25681(48.2)	36813(35.5)	0.2599	21454(45.5)	21502(45.6)	0.0020
Angiotensin II inhibitors	15452(29.0)	18862(18.1)	0.2570	12413(26.3)	12340(26.2)	0.0035
Diuretics	26229(49.2)	39619(38.2)	0.2239	22173(47.0)	22295(47.3)	0.0052
Vitamin D supplement	16968(31.8)	20682(19.9)	0.2745	14142(30.0)	11876(25.2)	0.1078
Vitamin E supplement	2647(4.9)	3896 (3.7)	0.0594	2233(4.7)	2270(4.8)	0.0037
Calcium channel blockers	15856(29.7)	24607(23.7)	0.1368	13187(28)	13812(29.3)	0.0294
Antihypertensives, other	11075(20.7)	15940(15.3)	0.1412	9182(19.4)	8919(18.9)	0.0142
Antihypertensive combinations	325(0.6)	293 (0.2)	0.0492	234(0.4)	232(0.4)	0.0006
**Diabetic medications,** n (%)						
Insulin	28667(53.8)	28199(27.2)	0.5637	23987(50.9)	15936(33.8)	0.3512
Glipizide	11358(21.3)	8798(8.4)	0.3666	9518(20.2)	4938(10.4)	0.2723
Sitagliptin	10964(20.5)	0(0)	0.7201	9113(19.3)	0(0)	0.6927
Empagliflozin	5763(10.8)	1536(1.4)	0.3963	4881(10.3)	940(1.9)	0.3529
Pioglitazone	4878(9.1)	2678(2.5)	0.2826	3960(8.4)	1677(3.5)	0.2054
Canagliflozin	3935(7.3)	913(0.8)	0.3314	3249(6.8)	599(1.2)	0.2872
Glyburide	3364(6.3)	3388(3.2)	0.1431	2781(5.9)	1942(4.1)	0.0817
Dapagliflozin	2498(4.6)	669 (0.6)	0.2531	2127(4.5)	400(0.8)	0.2284
Linagliptin	2539(4.7)	0(0)	0.3164	2008(4.2)	0(0)	0.2984
Repaglinide	702(1.3)	340 (0.3)	0.1098	556(1.1)	222 (0.4)	0.0784
Rosiglitazone	499(0.9)	476 (0.4)	0.0574	358(0.7)	353(0.7)	0.0012
Acarbose	334(0.6)	154 (0.1)	0.0771	161(0.3)	143(0.3)	0.0067
Nateglinide	271(0.5)	110 (0.1)	0.0728	104(0.2)	108(0.2)	0.0018
Ertugliflozin	210(0.3)	40 (0.03)	0.0766	69(0.1)	40(0.08)	0.0181
Alogliptin	452(0.8)	0(0)	0.1309	14(0.03)	0(0)	0.0244

Abbreviations: NAFLD, nonalcoholic fatty liver disease, GLP-1Ras, glucagon-like peptide-1 receptor agonists; SMD, standard mean difference; SD, standard deviation; BMI, body mass index; ACE, angiotensin-converting-enzyme.

ESM Table 3: Laboratory findings of new GLP-1A users vs matched new metformin users in patients with NAFLD and type 2 diabetes

Variables	Before propensity matching			After propensity matching		
	GLP-1A (N=53249)	**Metformin** (N=103667)	**SMD**	GLP-1A (N=47097)	**Metformin** (N=47097)	**SMD**
**Vital signs:** *mean ± SD*						
SBP, (mmHg)	130 ± 17.2	131 ± 18.4	0.0206	130 ± 17.2	131 ± 18.2	0.0249
DBP, (mmHg)	77.1 ± 11	76.9 ± 11.8	0.0178	77.2 ± 11	76.9 ± 11.7	0.0245
**Liver function test:** *mean ± SD*						
ALT, (U/L)	43.1 ± 44.6	45.4 ± 55.7	0.0462	43.8 ± 46.4	44.3 ± 46.4	0.0095
AST, (U/L)	34.4 ± 28.6	37.4 ± 46.5	0.0785	34.7 ± 29	36.6 ± 43.4	0.0519
T.Bil, (µmol/L)	9.82± 11.51	10.81± 12.66	0.0819	9.89± 10.35	10.43± 10.83	0.0509
ALP, (U/L)	90.1 ± 41.6	91.5 ± 50.6	0.0302	89.5 ± 41.3	91.4 ± 50.4	0.0406
Serum Albumin, (g/dL)	40.80 ± 4.91	40.40 ± 5.41	0.0716	40.90 ± 0.488	40.50 ± 5.33	0.0832
**Coagulation profile:** *mean ± SD*						
PT (sec)	12.50 ± 6.20	12.80 ± 5.30	0.0486	12.60 ± 5.74	12.90 ± 5.45	0.0563
INR	1.20 ± 1.90	1.20 ± 1.29	0.0019	1.19 ± 1.79	1.19 ± 1.15	0.0002
APTT (sec)	30.80 ± 11.60	31 ± 11.60	0.0184	30.60 ± 11	31.10 ± 11.90	0.0416
**Complete blood count:** *mean ± SD*						
Hemoglobin (g/L),	135 ± 18	135 ± 19.3	0.0200	136 ± 17.9	134 ± 19.0	0.0878
Platelets (10^9/L)	247 ± 82.30	242 ± 84.90	0.0573	246 ± 81.30	247 ± 84.10	0.0093
HCT, (Proportion of 1.0)	0.40 ± 0.07	0.40 ± 0.07	0.0043	0.40 ± 0.07	0.40 ± 0.07	0.0685
**Metabolic profile:** *mean ± SD*						
HbA1C(Proportion of total hemoglobin)	0.08 ± 0.02	0.07 ± 0.02	0.5325	0.08 ± 0.02	0.07 ± 0.02	0.4842
Fasting blood glucose, (mmol/L)	8.38± 3.45	7.21± 2.63	0.3801	8.27 ± 3.35	7.27± 2.66	0.3155
Creatinine, (µmol/L)	77.78± 291.27	71.52± 183.76	0.0269	77.01± 284.41	72.74± 186.05	0.0171
GFR (mL/min/1.73 m^2)	83.9 ± 28	85.3 ± 27.1	0.0503	84.7 ± 27.5	84.1 ± 27.3	0.0224
Total protein (g/L)	70.90 ± 10.10	70.60 ± 10.33	0.0245	70.80 ± 10.12	70.60 ± 10.28	0.0176
BUN, (mmol/L)	43.69 ±22.4	41.44 ± 20.3	0.1097	43.13 ± 21.51	42.57 ± 21.7	0.0271
**Cardiac markers,** *mean ± SD*						
LDH, (µkat/L)	4.16± 5.66	4.24± 4.96	0.0171	4.14± 5.78	4.31± 5.51	0.0276
**Inflammatory markers,** *mean ± SD*						
Ferritin (µg /L)	176 ± 334	233 ± 780	0.0966	177 ± 343	216 ± 695	0.0695
CRP (ug/L)	1.89 ± 3.97	2.13 ± 4.44	0.0560	1.82 ± 3.85	2.11 ± 4.41	0.0708
Urate (mmol/L)	351.53 ± 129.67	361.64± 155.84	0.0698	352.12 ± 1322.05	362.83 ± 0143.94	0.0714
ESR,(mm/h)	26.6 ± 22.8	26 ± 23.8	0.0240	25.6 ± 22.1	26.6 ± 23.9	0.0409
**Lipid profile:** *mean ± SD*						
CH(mmol/L)	4.43± 1.26	4.58± 1.29	0.1146	4.43± 1.25	4.58± 1.33	0.1151
TG(mmol/L)	2.41± 2.66	2.25± 2.54	0.0587	2.37± 2.61	2.32± 2.82	0.0168
LDL(mmol/L)	2.38± 0.98	2.54 ± 0.99	0.1666	2.39± 0.98	2.52± 1.00	0.1378
HDL (mmol/L)	1.06± 0.35	1.09 ± 0.39	0.0729	1.06± 0.35	1.08± 0.39	0.0459

Abbreviations: NAFLD, nonalcoholic fatty liver disease; GLP-1RA, glucagon-like peptide-1 receptor agonists; SMD, standard mean difference; SD, standard deviation; SBP, systolic blood pressure; DBP, diastolic blood pressure; ALT, alanine transaminase ; AST, aspartate aminotransferase; T.Bil, total bilirubin;ALP, alkaline phosphatase; PT, prothrombin time; INR, international normalised ratio; APTT, activated partial thromboplastin; HCT, hematocrit; HbA1C, hemoglobin A1C ; BUN, blood urea nitrogen; GFR, glomerular filtration rate ; LDH, lactate dehydrogenase; CRP, ; CH, cholesterol; TG, triglycerides; LDL, low-density lipoprotein ; HDL, high-density lipoprotein.

ESM Table 4: Baseline characteristics of new GLP-1RA users vs matched new Second- or- third-line drug users in patients with NAFLD and type 2 diabetes

Variables	Before propensity score match			After propensity score match		
	**GLP-1RA** (N=53249)	**Second or third-line drugs** (N=2638687)	**SMD**	**GLP-1RA** (N=52166)	**Second – or third-line drugs** (N=52166)	**SMD**
**Age in years, mean(SD)**	54.6 ± 12	59.6 ± 15.3	0.3599	54.7 ± 12	54.5 ± 13.3	0.0096
**Sex, n (%),** *Female*	32188(60.4)	1285308(48.7)	0.2374	31398(60.1)	32018(61.3)	0.0243
**Ethnicity, n (%)**						
Hispanic or Latino	5161(9.6)	211021(7.9)	0.0597	4971(9.5)	4466(8.5)	0.0338
**Race, n (%)**						
White	38990(73.2)	1627406(61.6)	0.2483	38138(73.1)	39096(74.9)	0.0419
Black or African Americans	7649(14.3)	551430(20.8)	0.1721	7520(14.4)	6924(13.2)	0.0331
Others	501(9.9)	381655(14.4)	0.1380	5216(9.9)	4950(9.4)	0.0172
**Nicotine dependence,** n (%)	9492(17.8)	180263(6.8)	0.3392	9129(17.5)	8802(16.8)	0.0166
**BMI (kg/m^2),** mean ±SD	37.1 ± 6.85	32.7 ± 7.28	0.6240	37.1 ± 6.85	34.8 ± 7.51	0.3129
**Comorbidities,** n (%)						
Hypertension	40674(76.3)	908962(34.4)	0.9306	39599(75.9)	39688(76.0)	0.0040
Hyperlipidemia	32759(61.5)	532465(20.1)	0.9270	31712(60.7)	31764(60.8)	0.0020
Chronic lower respiratory diseases	17894(33.6)	286857(10.8)	0.5683	17172(32.9)	17082(32.7)	0.0037
Hypercholesterolemia	12968(24.3)	208027(7.8)	0.4596	12490(23.9)	10350(19.8)	0.0993
Diabetic polyneuropathy	9289(17.4)	67781(2.5)	0.5117	8342(15.9)	7770(14.8)	0.0303
Chronic kidney disease	7534(14.1)	189532(7.1)	0.2271	7136(13.6)	6476(12.4)	0.0376
Peripheral vascular diseases	4094(7.6)	82925(3.1)	0.2019	3855(7.3)	3416(6.5)	0.0331
Diabetic retinopathy	3629(6.8)	45682(1.7)	0.2534	3151(6.0)	2761(5.2)	0.0323
Diabetic nephropathy	3795(7.1)	25902(0.9)	0.3154	3164(6.0)	2934(5.6)	0.0188
Glomerular diseases	1841(3.4)	25297(0.9)	0.1707	1554(2.9)	2064(3.9)	0.0535
Cirrhosis of liver	4017(7.5)	30563(1.1)	0.3169	923(7.5)	917(1.7)	0.2766
Osteoporosis	2514(4.7)	54262(2.0)	0.1477	2418(4.6)	2361(4.5)	0.0052
Obstructive sleep apnea	18993(35.6)	182996(6.9)	0.7289	17971(34.4)	18175(34.8)	0.0078
**Cardiovascular medications,** n (%)						
Beta-blockers	23660(44.4)	554644(21.0)	0.5153	22863(43.8)	22156(42.4)	0.0274
Antiarrhythmics	25198(47.3)	429457(16.2)	0.7071	24405(46.7)	17824(34.1)	0.2592
Antilipemic agents	36367(68.2)	727957(27.5)	0.8923	35301(67.6)	35324(67.7)	0.0009
ACE inhibitors	25681(48.2)	493502(18.7)	0.6588	24759(47.4)	24473(46.9)	0.0110
Angiotensin II inhibitors	15452(29.0)	276912(10.4)	0.4784	14850(28.4)	14924(28.6)	0.0031
Diuretics	26229(49.2)	557264(21.1)	0.6166	25408(48.7)	24081(46.1)	0.0510
Vitamin D supplement	16968(31.8)	244847(9.2)	0.5819	16318(31.2)	12040(23.0)	0.1851
Vitamin E supplement	2647(4.9)	37947(1.4)	0.2016	2543(4.8)	1927(3.6)	0.0583
Calcium channel blockers	15856(29.7)	376978(14.2)	0.3805	15281(29.2)	15346(29.4)	0.0027
Antihypertensives, other	11075(20.7)	239088(9.0)	0.3339	10632(20.3)	9971(19.1)	0.0318
Antihypertensive combinations	325(0.6)	7715(0.2)	0.0474	313(0.6)	254(0.4)	0.0154
**Diabetic medications,** n (%)						
Metformin	39159(73.5)	581679(22.0)	1.2030	38200(73.2)	26183(50.1)	0.4878
Insulin	28667(53.8)	367637(13.9)	0.9297	27679(53.0)	8652(16.5)	0.8287
Glipizide	11358(21.3)	90754(3.4)	0.5643	10943(20.9)	1433(2.7)	0.5876
Sitagliptin	11075(20.7)	239088(9.0)	0.3339	10632(20.3)	9971(19.1)	0.0318
Empagliflozin	5763(10.8)	17033(0.6)	0.4486	5604(10.7)	374(0.7)	0.4418
Pioglitazone	4878(9.1)	38826(1.4)	0.3479	4600(8.8)	729(1.3)	0.3420
Canagliflozin	3935(7.3)	10725(0.4)	0.3668	3807(7.2)	231(0.4)	0.3611
Glyburide	3364(6.3)	33261(1.2)	0.2672	3192(6.1)	532(1.0)	0.2775
Dapagliflozin	2498(4.6)	9232(0.3)	0.2797	2406(4.6)	221(0.4)	0.2698
Linagliptin	2539(4.7)	19514(0.7)	0.2481	2423(4.6)	846(1.6)	0.1742
Repaglinide	702(1.3)	6928(0.2)	0.1194	674(1.2)	251(0.4)	0.0866
Rosiglitazone	499(0.9)	4045(0.1)	0.1066	343(0.6)	237(0.4)	0.0273
Acarbose	334(0.6)	2902(0.1)	0.0854	283(0.5)	242(0.4)	0.0111
Nateglinide	271(0.5)	3148(0.1)	0.0697	228(0.4)	202(0.3)	0.0078
Ertugliflozin	210(0.3)	672(0.02)	0.0807	154(0.2)	166(0.3)	0.0042
Alogliptin	452(0.8)	2172(0.08)	0.1128	376(0.7)	376(0.7)	< 0.0001

Abbreviations: NAFLD, nonalcoholic fatty liver disease; GLP-1RAs, glucagon-like peptide-1 receptor agonists; SMD, standard mean difference; SD, standard deviation; BMI, body mass index; ACE, angiotensin-converting-enzyme.

ESM Table 5: Laboratory findings of new GLP-1RA users vs matched new Second -or third-line drugs users in patients with NAFLD and type 2 diabetes

Variables	Before propensity matching			After propensity matching		
	**GLP-1RA** (N=53249)	**Second- or third-line drugs** (N=2638687)	**SMD**	**GLP-1RA** (N=52166)	**Second or third-line drugs** (N=52166)	**SMD**
**Vital signs:** *mean ± SD*						
SBP, mmHg	130 ± 17.2	133 ± 20.3	0.1248	130 ± 17.2	131 ± 18.6	0.0402
DBP, mmHg	77.1 ± 11	75.9 ± 12.2	0.1019	77.1 ± 11	77.1 ± 11.6	0.0026
**Liver function test:** *mean ± SD*						
ALT (U/L)	43.1 ± 44.6	31.5 ± 47	0.2524	43.3 ± 44.9	33.7 ± 70.9	0.1604
AST (U/L)	34.4 ± 28.6	28.4 ± 49.5	0.1498	34.5 ± 28.7	28.2 ± 23.8	0.2398
T.Bil (µmol/L)	9.82 ± 11.51	10.02± 12.35	0.0172	9.82± 9.95	9.37± 6.72	0.0533
ALP (U/L)	90.1 ± 41.6	89 ± 49	0.0226	89.8 ± 41.5	87.1 ± 45.8	0.0606
Serum Albumin (g/dL)	40.80 ± 4.91	40.40 ± 5.41	0.0716	40.90 ± 0.488	40.50 ± 5.33	0.0832
**Coagulation profile:** *mean ± SD*						
PT (sec)	12.50 ± 6.20	13.40 ± 6.20	0.1327	12.60 ± 5.99	12.80 ± 6.26	0.0406
INR	1.20 ± 1.90	1.25 ± 1.35	0.0306	1.20 ± 1.92	1.20 ± 1.2 0	0.0007
APTT (sec)	30.80 ± 11.60	31.10 ± 12	0.0261	30.80 ± 11.50	31.1 0± 12.10	0.0297
**Complete blood count:** *mean ± SD)*						
Hemoglobin (g/L),	135 ± 18	131 ± 20.5	0.2318	135 ± 17.9	133 ± 19.2	0.1149
Platelets (10^9/L)	247 ± 82.3	246 ± 84.2	0.0138	247 ± 82.4	256 ± 80.5	0.1118
HCT, Proportion of 1.0.	40.4 ± 6.68	39.1 ± 6.77	0.1892	40.4 ± 6.7	39.8 ± 6.45	0.0840
**Metabolic profile:** *mean ± SD*						
HbA1C(Proportion of total hemoglobin)	0.08 ± 0.0	0.08 ± 0.02	0.3131	0.08 ± 0.02	0.08 ± 0.02	0.4064
Fasting blood glucose (mmol/L /)	8.38 ± 3.46	7.71 ± 3.12	0.2084	8.32 ± 3.41	7.44 ± 2.84	0.2841
Creatinine (µmol/L)	77.78 ± 292.04	89.21 ± 166.23	0.0479	77.78 ± 287.46	83.88 ± 206.64	0.0257
GFR (mL/min/1.73 m^2)	83.9 ± 28	78.5 ± 32	0.1822	84.2 ± 27.8	80.7 ± 29.5	0.1217
Total protein (g/L)	70.90 ± 10.10	70.50 ± 10.00	0.0302	70.80 ± 10.10	70.70 ± 10.10	0.0136
BUN (mmol/L)	43.69 ± 22.4	51.25 ± 32.2	0.2648	43.41 ± 21.84	47.05 ± 28.28	0.1421
**Cardiac markers,** *mean ± SD*						
LDH, (µkat/L)	4.16 ± 5.66	4.49 ± 5.71	0.0586	4.14 ± 5.64	4.44 ± 7.06	0.0453
**Inflammatory markers,** *mean ± SD*						
Ferritin, (µg /L)	176 ± 334	269 ± 845	0.1460	176 ± 336	205 ± 496	0.0677
CRP (ug/L)	1.89 ± 3.97	2.33 ± 4.68	0.1003	1.87 ± 3.94	1.91 ± 4.04	0.0110
Urate(mmol/L)	351.53 ± 129.67	367.59 ± 140.97	0.1166	351.53 ± 129.67	363.42± 132.64	0.0893
ESR (mm/h)	26.6 ± 22.8	28.8 ± 26.3	0.0911	26.3 ± 22.6	26.4 ± 23.9	0.0037
**Lipid profile:** *mean ± SD*						
CH (mmol/L)	4.43± 1.26	4.48± 1.24	0.0262	4.43± 1.26	4.58± 1.26	0.1118
TG (mmol/L)	2.41± 2.66	1.97± 1.93	0.1877	2.40± 2.66	2.18± 1.98	0.0914
LDL (mmol/L)	2.38± 0.98	2.47± 0.98	0.0991	2.38± 0.98	2.52± 0.98	0.1470
HDL (mmol/L)	1.06± 0.35	1.12± 0.44	0.1513	1.06 ± 0.36	1.12± 0.40	0.1514

Abbreviations: NAFLD, nonalcoholic fatty liver disease; GLP-1RA, glucagon-like peptide-1 receptor agonists; SMD, standard mean difference; SD, standard deviation; SBP, systolic blood pressure; DBP, diastolic blood pressure; ALT, alanine transaminase ; AST, aspartate aminotransferase; T.Bil, total bilirubin;ALP, alkaline phosphatase; PT, prothrombin time; INR, international normalised ratio; APTT, activated partial thromboplastin; HCT, hematocrit; HbA1C, hemoglobin A1C ; BUN, blood urea nitrogen; GFR, glomerular filtration rate ; LDH, lactate dehydrogenase; CRP, ; CH, cholesterol; TG, triglycerides; LDL, low-density lipoprotein ; HDL, high-density lipoprotein.

ESM Table 6: Sensitivity analysis to assess the cardiovascular outcomes and all cause of mortality between the new users of GLP-1RA vs. SGLT-2 inhibitors in patients with NAFLD and type 2 diabetes after excluding index events within 2 years after the index date

Outcomes	GLP-1RA (N=38804), n	SGLT-2 inhibitors (N=38804), n	HR (95% CI)
**Primary outcome**			
Heart failure	857	745	0.962 (0.871,1.062
MACE	659	542	0.992 (0.885,1.113)
Cerebrovascular Diseases	1,052	875	0.989 (0.904,1.083)
**Secondary outcome**			
All-cause mortality	745	563	1.087 (0.973,1.214)

Abbreviations: GLP-1RA, glucagon-like peptide-1 receptor agonists; SGLT-2 sodium-glucose cotransporter-2; HR, hazard ratio; MACE, Major adverse cardiovascular events; NAFLD, nonalcoholic fatty liver disease.

MACE as a composite endpoint was defined as the first occurrence of unstable angina, or myocardial infarction, and revascularization, including percutaneous coronary intervention or coronary artery bypass graft. A composite of cerebrovascular disease was defined as the first occurrence of stroke (ischemic or hemorrhagic stroke), cerebral infarction, transient ischemic attack, carotid intervention, or surgery.

ESM Table 7: Sensitivity analysis to assess the cardiovascular outcomes and all cause of mortality between the new users GLP-1RA vs. Metformin in patients with NAFLD and type 2 diabetes after excluding index events within 2 years after the index date.

**Outcomes**	**GLP-1RA** (N=47097), n	**Metformin** (N=47097), n	**HR (95% CI)**
**Primary outcome**			
Heart failure	1815	1521	1.10 (1.02, 1.17)
Cerebrovascular Diseases	2103	1919	1.00 (0.94, 1.07)
MACE	1255	1142	0.98 (0.91, 1.07)
**Secondary outcome**			
Mortality	1336	1148	1.05 (0.97, 1.14)

Abbreviations: GLP-1RA, glucagon-like peptide-1 receptor agonists; SGLT-2 sodium-glucose cotransporter-2; HR, hazard ratio; MACE, Major adverse cardiovascular events; NAFLD, nonalcoholic fatty liver disease.

MACE as a composite endpoint was defined as the first occurrence of unstable angina, or myocardial infarction, and revascularization, including percutaneous coronary intervention or coronary artery bypass graft. A composite of cerebrovascular disease was defined as the first occurrence of stroke (ischemic or hemorrhagic stroke), cerebral infarction, transient ischemic attack, carotid intervention, or surgery.

ESM Table 8: Sensitivity analysis to assess the cardiovascular outcomes and all cause of mortality between the new users GLP-1RA vs. Second- or third line antidiabetic medications in patients with NAFLD and type 2 diabetes after excluding index events within 2 years after the index date.

Outcomes	GLP-1RA (N=52166), n	Second- or third-line drugs (N=52166), n	HR (95%CI)
**Primary outcome**			
Heart failure	2092	3423	0.90 (0.85, 0.95)
Cerebrovascular Diseases	2493	3602	1.02 (0.97, 1.08)
MACE	1461	2434	0.90 (0.84, 0.96)
**Secondary outcome**			
Mortality	1524	3476	0.70 (0.66, 0.74)

Abbreviations: GLP-1RA, glucagon-like peptide-1 receptor agonists; SGLT-2 sodium-glucose cotransporter-2; HR, hazard ratio; MACE, Major adverse cardiovascular events; NAFLD, nonalcoholic fatty liver disease.

MACE as a composite endpoint was defined as the first occurrence of unstable angina, or myocardial infarction, and revascularization, including percutaneous coronary intervention or coronary artery bypass graft. A composite of cerebrovascular disease was defined as the first occurrence of stroke (ischemic or hemorrhagic stroke), cerebral infarction, transient ischemic attack, carotid intervention, or surgery.
